# Biosynthetic Strategies of Berberine Bridge Enzyme-like Flavoprotein Oxidases toward Structural Diversification in Natural Product Biosynthesis

**DOI:** 10.1021/acs.biochem.4c00320

**Published:** 2024-08-12

**Authors:** Gwen Tjallinks, Andrea Mattevi, Marco W. Fraaije

**Affiliations:** † Biomolecular Sciences and Biotechnology Institute, University of Groningen, Groningen 9747 AG, The Netherlands; ‡ Department of Biology and Biotechnology, University of Pavia, Pavia 27100, Italy

**Keywords:** natural product biosynthesis, enzyme mechanism, oxidoreductase, flavoprotein, FAD-linked oxidase, vanillyl-alcohol oxidase, berberine bridge-like oxidase

## Abstract

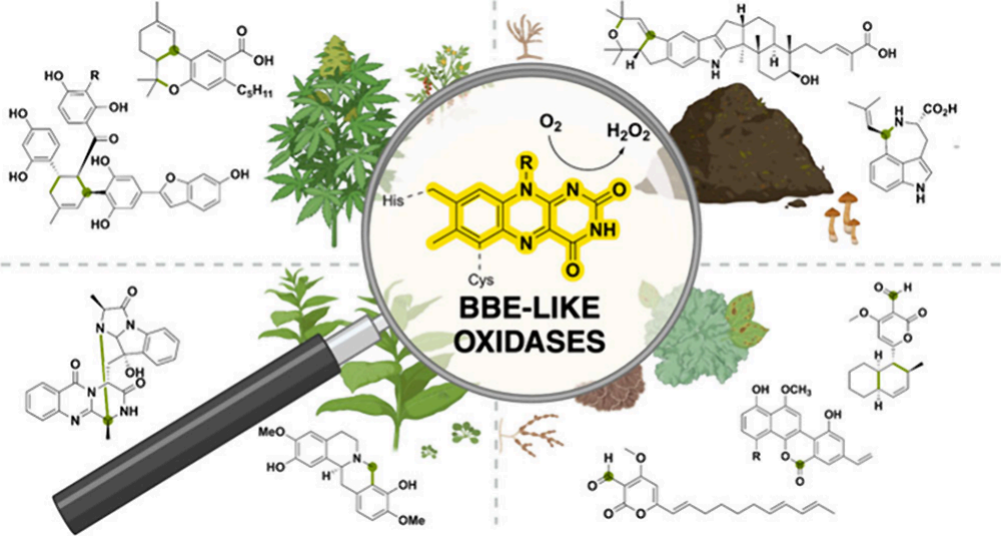

Berberine bridge enzyme-like oxidases are often involved in natural product biosynthesis and are seen as essential enzymes for the generation of intricate pharmacophores. These oxidases have the ability to transfer a hydride atom to the FAD cofactor, which enables complex substrate modifications and rearrangements including (intramolecular) cyclizations, carbon–carbon bond formations, and nucleophilic additions. Despite the diverse range of activities, the mechanistic details of these reactions often remain incompletely understood. In this Review, we delve into the complexity that BBE-like oxidases from bacteria, fungal, and plant origins exhibit by providing an overview of the shared catalytic features and emphasizing the different reactivities. We propose four generalized modes of action by which BBE-like oxidases enable the synthesis of natural products, ranging from the classic alcohol oxidation reactions to less common amine and amide oxidation reactions. Exploring the mechanisms utilized by nature to produce its vast array of natural products is a subject of considerable interest and can lead to the discovery of unique biochemical activities.

Natural products exhibit astonishing structural diversity and this molecular variety results in a vast array of biological activities.^1^ Nevertheless, secondary metabolites generally originate from a small number of starting components, which are obtained from primary metabolic pathways. Next to core biosynthetic enzymes, tailoring enzymes are required to enhance the complexity of natural products.^2−5^ There are a multitude of biocatalysts involved in the modification of the metabolite precursors including oxidoreductases, halogenases, (acyl-, glycosyl-)transferases and ligases.^6^

In particular, oxidoreductases are intriguing since complex biochemical transformations often require changes in redox states.^7,8^ Therefore, oxidoreductases are essential players in the complex pathways that lead to the synthesis of secondary metabolites in different organisms. Oxidation reactions such as dehydrogenation, epoxidation and hydroxylation are typical redox reactions in natural product biosynthesis.^9^ Flavin-dependent oxidoreductases are able to perform these transformations and are therefore commonly observed in secondary metabolic pathways. This Review focuses on flavin-dependent oxidases from the vanillyl-alcohol oxidase/*p*-cresol methylhydroxylase (VAO/PCMH) family.^10^ We will specifically cover the berberine bridge enzyme (BBE) subfamily since they are often involved in natural product biosynthesis and seen as essential enzymes for the generation of intricate pharmacophores.^11−13^

## VAO/PCMH Flavoprotein Family

Based on structural data and sequence homology there are six different families of flavin-dependent oxidases.^14^ One of these six families is the VAO/PCMH flavoprotein family, named after the fungal vanillyl-alcohol oxidase and bacterial *p*-cresol methylhydroxylase, feature a distinct flavin adenine dinucleotide (FAD) binding domain in the N-terminal portion of the protein.^12,15^ This conserved domain allows for the binding of the adenosine diphosphate and ribityl functional groups of FAD. Except for the flavin-binding domain, VAO-type enzymes contain a substrate-binding domain (cap domain) with greater variability positioned above the isoalloxazine ring of the cofactor. This structural arrangement enables significant diversity in the active site structures and, consequently, in the catalytic activities of these enzymes.^11^

The crystal structure of VAO was the first to show FAD covalently linked to the protein via an 8α-*N*^3^-histidyl–FAD linkage.^16−18^ In the last few decades, many more members of the VAO family have been identified and shown to contain a covalently tethered flavin cofactor.^12^ Intriguingly, a significant number of them even had the flavin cofactor bicovalently bound to the protein, such as glucooligosaccharide oxidase (GOOX).^19,20^ Bicovalently tethering of the FAD enables flavoproteins to have a rather open active site thereby allowing them to accept larger substrates such as secondary metabolites but also oligosaccharides.^21−23^ Another effect of covalently binding FAD is the increase in flavin redox potential, which is highest for bicovalently bound flavoproteins.^24^ Increasing the redox potential enhances the oxidative power of the flavin cofactor making them particularly adept at catalyzing demanding oxidation reactions.

**Scheme 1:**
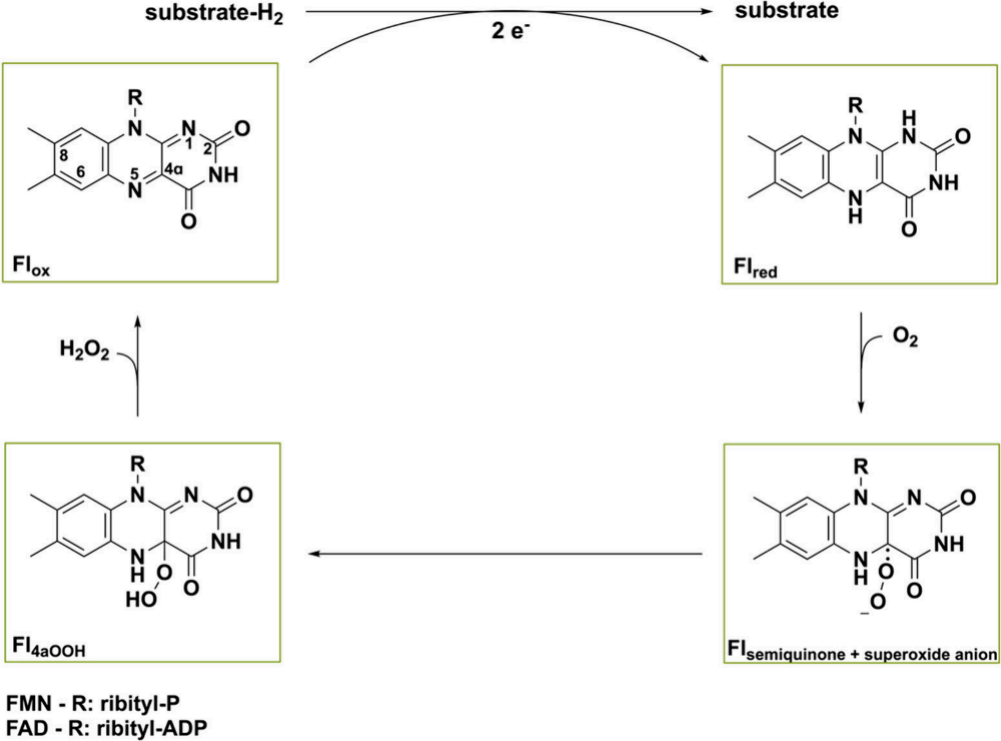
Reductive and Oxidative Half Reaction of Flavoprotein Oxidases First, the flavin is reduced by the substrate in the reductive half reaction generating Fl_red_. Then, Fl_red_ reacts with O_2_ via a single-electron transfer to produce Fl_semiquinone_ and recombination with the superoxide anion results in Fl_C4aOOH_. Oxidases release H_2_O_2_ to obtain Fl_ox_ again.

Flavoprotein oxidases typically contain FAD, and sometimes flavin mononucleotide (FMN), as prosthetic group for mediating redox reactions.^9^ Reactions are catalyzed in a two-step manner, consisting of a reductive and oxidative half reaction (Scheme 1).^25^ In the former half, the substrate is oxidized while the flavin is being reduced by hydride attack on the N5-atom. In the subsequent oxidative half reaction, the reduced flavin (Fl_red_) returns to its oxidized state (Fl_ox_) as the cosubstrate molecular oxygen (O_2_) gets reduced. The first step in the oxidative half reaction is the single electron transfer of Fl_red_ to O_2_ producing a flavin semiquinone and superoxide species that can be covalently tethered yielding the C4a-hydroperoxyflavin species (Fl_4aOOH_).^26^ This species can then undergo multiple dissociation pathways, leading to different reactivities, making flavins very versatile organic cofactors. Oxidases typically use elimination and proton transfer to release hydrogen peroxide (H_2_O_2_) and thereby regenerate Fl_ox_. The elevated redox potential of (bi)covalently bound flavoprotein oxidases causes O_2_ to be one of the few electron acceptors that they can employ. Hence, in order to regenerate the reduced cofactor, oxidases use O_2_ as an electron acceptor by definition, effectively rendering the reaction irreversible.

## Berberine Bridge Enzyme Subfamily

**Figure 1:**
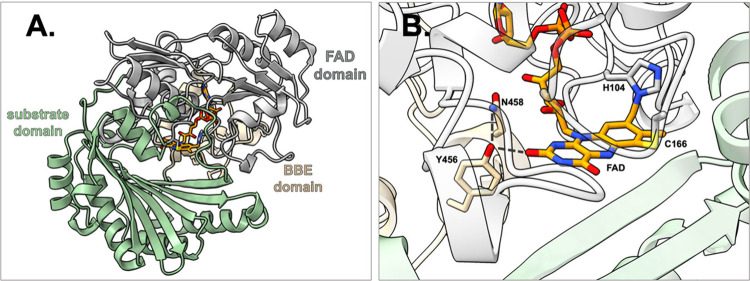
(a) The structure of *Ec*BBE (PDB: 3D2J) with the FAD-domain in silver, the substrate-domain in dark sea green and the BBE-domain in moccasin. The FAD-cofactor is colored orange, and all atoms are shown as ball-and-stick models. (b) The FAD-binding site of *Ec*BBE featuring the distinctive C-terminal Y/FxN motif. The FAD cofactor is bicovalently bound to residues Cys166 and His104.

Within the VAO/PCMH flavoprotein family, there are 11 different subfamilies out of which one has members involved in secondary metabolite biosynthesis in both plants and microorganisms.^23^ These are named BBE-like enzymes and constitute a sizable portion of the VAO-fold oxidoreductase family. Apart from the common FAD- and substrate-binding domains, they have a distinct structural characteristic near the FAD-binding site which serves as a distinguishing feature of the BBE subfamily (Figure 1a).^27^ This structural characteristic has been annotated as a domain (pfam entry PF08031) and contains a special C-terminus with Y/FxN motif that, in the case of a Tyr, creates a specific hydrogen bonding network proximal to the isoalloxazine ring of FAD and conserved Asn residue (Figure 1b). This motif helps shape the O_2_ binding pocket and affects the positioning of the ribityl moiety of FAD.^28^

**Scheme 2:**
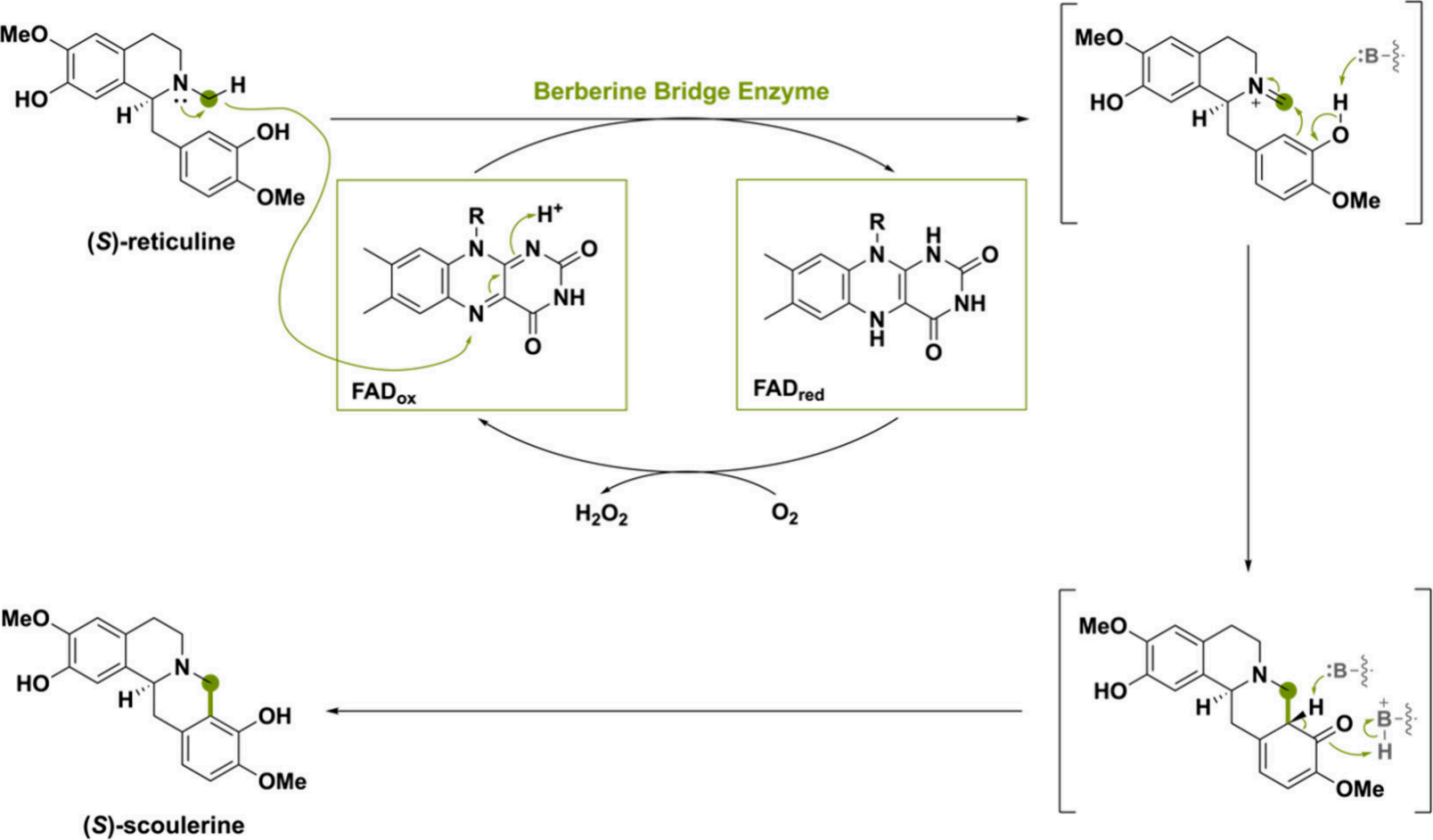
Oxidative Ring Closure Reaction from (*S*)-Reticuline to (*S*)-Scoulerine Catalyzed by the Plant-Derived Berberine Bridge Enzyme The green dot indicates where the hydride is getting transferred to the N5 of the FAD cofactor.

The (*S*)-reticuline oxidase, commonly known as the berberine bridge enzyme (BBE), from the plant *Eschscholzia californica* is the name-bearer for this subfamily and catalyzes the conversion of (*S*)-reticuline to (*S*)-scoulerine by mediating an oxidative ring closure reaction (Scheme 2). This reaction is proposed to occur through a stepwise oxidation of the *N*-methyl group to the iminium ion followed by a Friedel–Crafts acylation.^29^ (*S*)-Reticuline is the source of a wide range of benzylisoquinoline alkaloids (BIA) metabolites in secondary plant metabolism.^30,31^ A branch point in the biosynthesis of BIAs is marked by the C–C bond created by action of BBE, which is known as the berberine bridge.^32^ BBE-like flavoproteins typically contain a bicovalently bound FAD. However, there are examples of BBE-like oxidases with monocovalently bound FAD and singular instances where there is no covalent linkage with FAD whatsoever.^33−36^

## Unconventional Oxidation Reactions Catalyzed by BBE-like Oxidases

Even though BBE-like oxidases are named as such, they are functionally very different from the original name-bearer of this subfamily. They are capable of catalyzing a wide range of different reactions not only limited to the berberine bridge formation but also seen as essential enzymes for the generation of a plethora of intricate pharmacophores. A common trait among these unconventional oxidases is their capacity to generate novel C–C, C–O, or C–N bonds.^35,37−40^ These BBE-like oxidases promote the rearrangement of the molecular skeleton by catalyzing carbon-heteroatom and carbon–carbon bond oxidations. Oxidation can lead to the formation of new intramolecular bonds or prime the substrate to undergo hydration or dimerization reactions. These redox reactions have been of widespread interest in the field of synthetic electrochemisty^41−43^ and here we show the clever ways that nature utilizes BBEs to accomplish such challenging transformations. Even though BBE-like oxidases perform diverse activities, the exact mechanistic functioning of the enzymes performing the reactions is not always known. In this Review, we will go over multiple examples of experimentally validated BBE-like oxidoreductases in which the complexity of these biotransformations is portrayed.

**Scheme 3:**
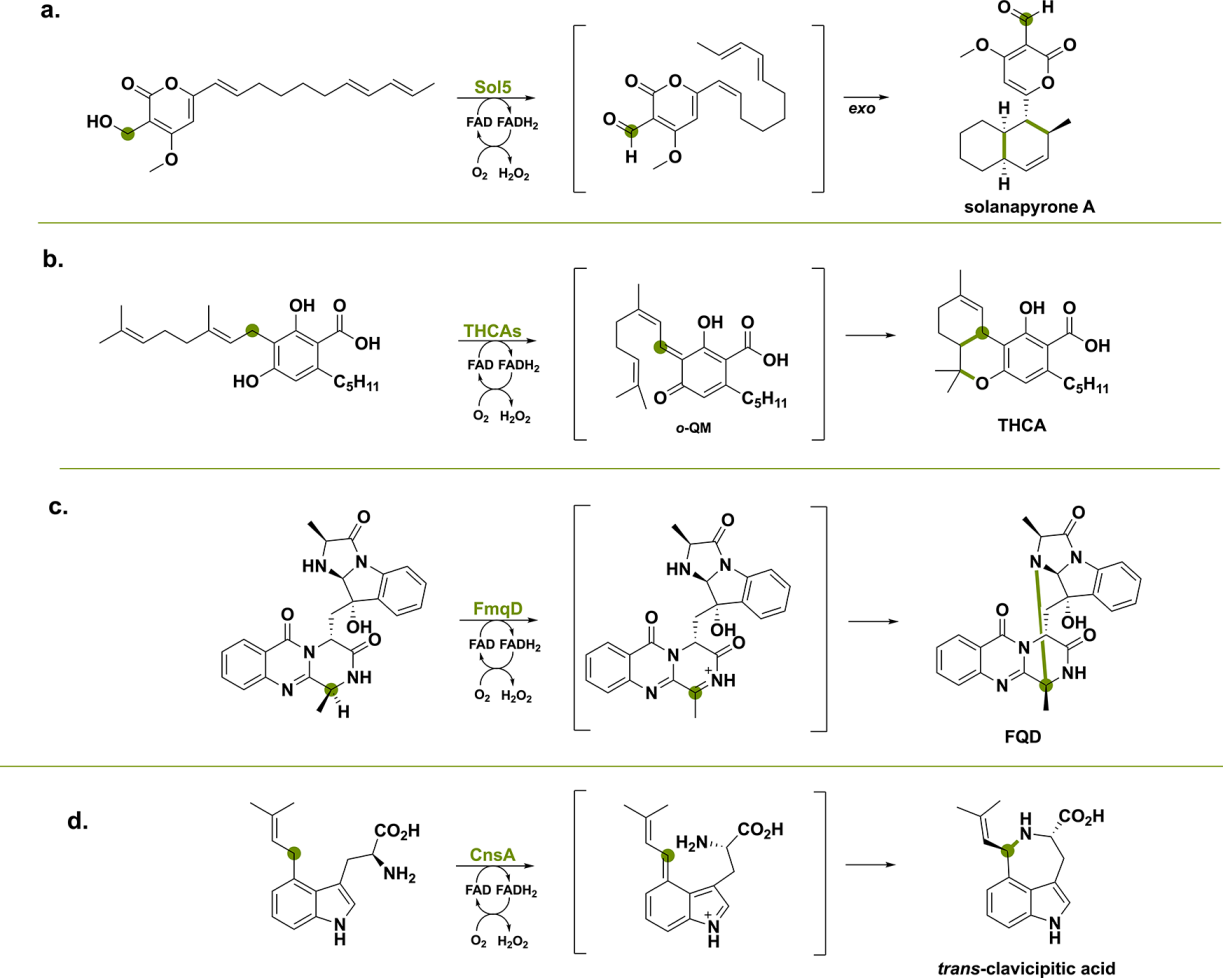
Four Modes of Action Illustrated by BBE-like Enzymes with Examples Taken from Literature (a) Aromatic alcohol oxidation catalyzed by Sol5 leading to a Diels-Alder cycloaddition reaction. (b) Oxidative cyclization reaction catalyzed by THCA synthase via an *ortho*-quinone methide intermediate. (c) Amide oxidation reaction catalyzed by FmqD with concomitant intramolecular cyclization. (d) Oxidative cyclization reaction catalyzed by CnsA. The green dot indicates where the hydride is getting transferred to the N5 of the FAD cofactor.

Below we highlight four generalized modes of action by which BBE-like oxidases enable the synthesis of intricate natural products. In Scheme 3 an example reaction is given for each reaction type that BBE-like oxidases utilize in natural product biosynthesis. The first reaction type is classical alcohol oxidation, where hydride transfer occurs at the α-carbon atom. The example shown in Scheme 3a illustrates a simple alcohol oxidation causing the dienophile to become sufficiently electron deficient and therefore allowing a [4 + 2] cycloaddition.^44^ The second example is the deprotonation of a phenol-derivative which promotes the hydride transfer at a distant carbon atom (Scheme 3b).^45^ This is similar to the benzylic oxidation reaction catalyzed by the classic VAO, with the only difference being the *ortho*-orientation of the hydroxy group compared to the usual *para*-orientation. Hydride transfer is achieved via the generation of an *ortho*-quinone methide (ο-QM) intermediate, that can then undergo different reactions such as the [4 + 2] cycloaddition leading to THCA. This ο-QM intermediate is observed in many BBE-like oxidases some of which have already been discussed by Purdy et al.^46^ The third example is the oxidation of a carbon–nitrogen bond, leading to hydride transfer from the α-carbon atom and thereby generating a transient imine or iminium cation that can undergo derivatization similar to the berberine bridge enzyme (Scheme 3c).^47^ The last example reaction is the hydride transfer from monoterpene indole alkaloids (Scheme 3d).^48^ The generated intermediate, stabilized via delocalized electrons from the indole *N*-atom, can undergo a multitude of derivatizations, including intramolecular cyclization but also water addition with concomitant oxidative cyclization. A phylogenetic tree was made but did not reveal clustering of the BBE-like enzymes into separate clades according to their function. The different activities might have evolved independently over time and other factors such as the enzyme’s origin and the type of natural product produced could be a reason for this.

### Alcohol Oxidation

Flavin-dependent oxidases are particularly known for the oxidation of alcohol groups. Examples include the methanol oxidases from methylotrophic yeasts^49−51^ and glucose oxidases^52^ secreted by filamentous fungi. Below we elaborate on alcohol oxidations occurring in biosynthetic pathways that can facilitate subsequent noteworthy transformations.

**Scheme 4:**
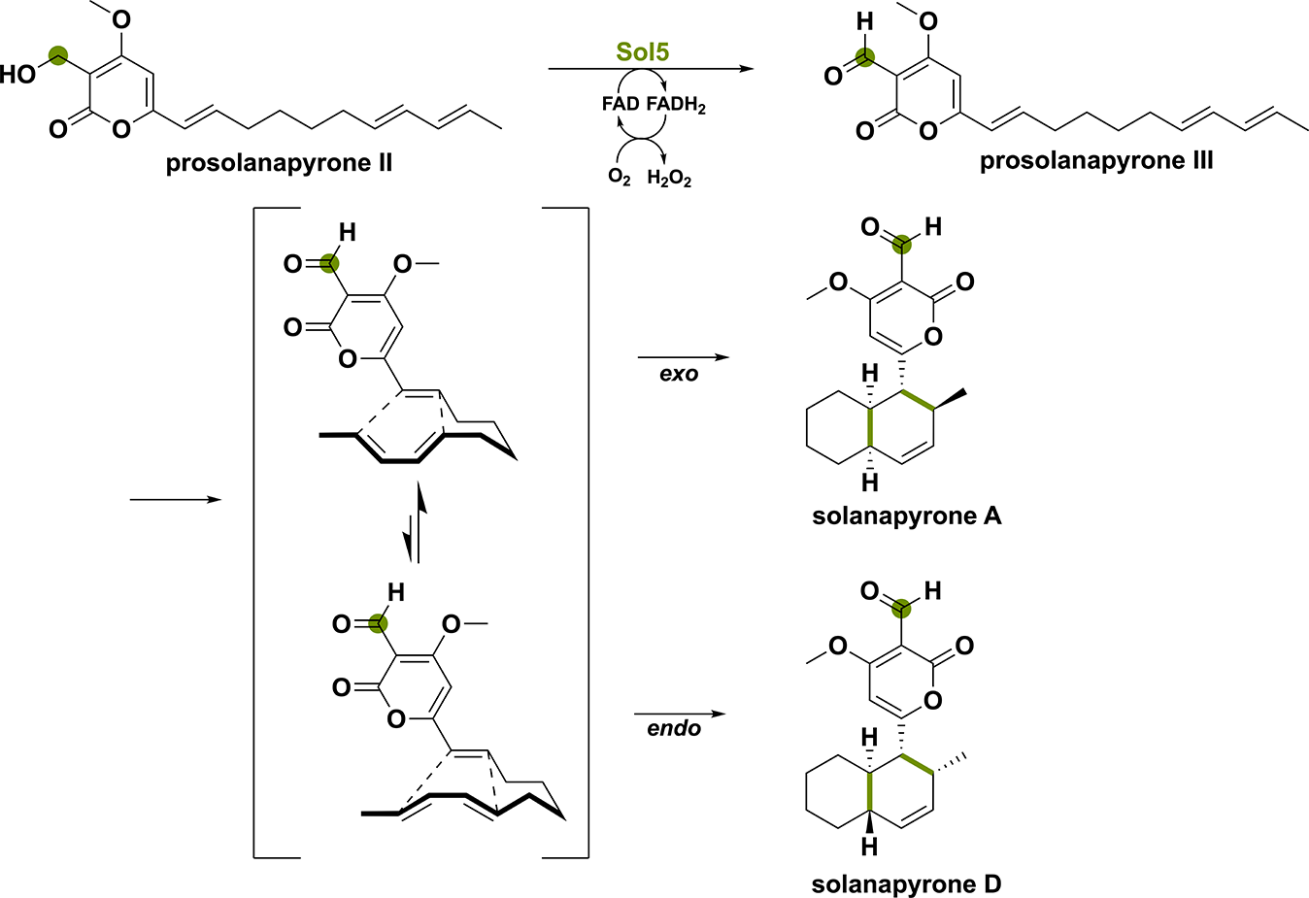
[4 + 2] Cyclization Enabled by the Alcohol Oxidation of Prosolanapyrone II to Aldehyde Mediated by the FAD-Enzyme Sol5

The first example concerns a BBE-like oxidase found in *Alternaria solani*, a pathogenic fungus that causes early blight in tomato and potato plants.^53^ Numerous polyketides are produced by this fungus including solanapyrone, which has a decalin structure formed through a [4 + 2] cycloaddition comparable to lovastatin skeleton formation.^35,54^ Initially assuming that a polyketide synthase would be responsible for the cycloaddition reaction as seen with lovastatin, it turned out that the flavoprotein oxidase Sol5 was the enzyme involved.^44^ Although this flavoenzyme performs a single oxidation of a primary alcohol group of prosolanapyrone II to the corresponding aldehyde, it also lowers the LUMO energy of the dienophile, thereby promoting the [4 + 2] cycloaddition (Scheme 4). Hence, the initial redox reaction performed by the BBE-like oxidase Sol5 is making this cycloaddition possible.^55^

**Scheme 5:**
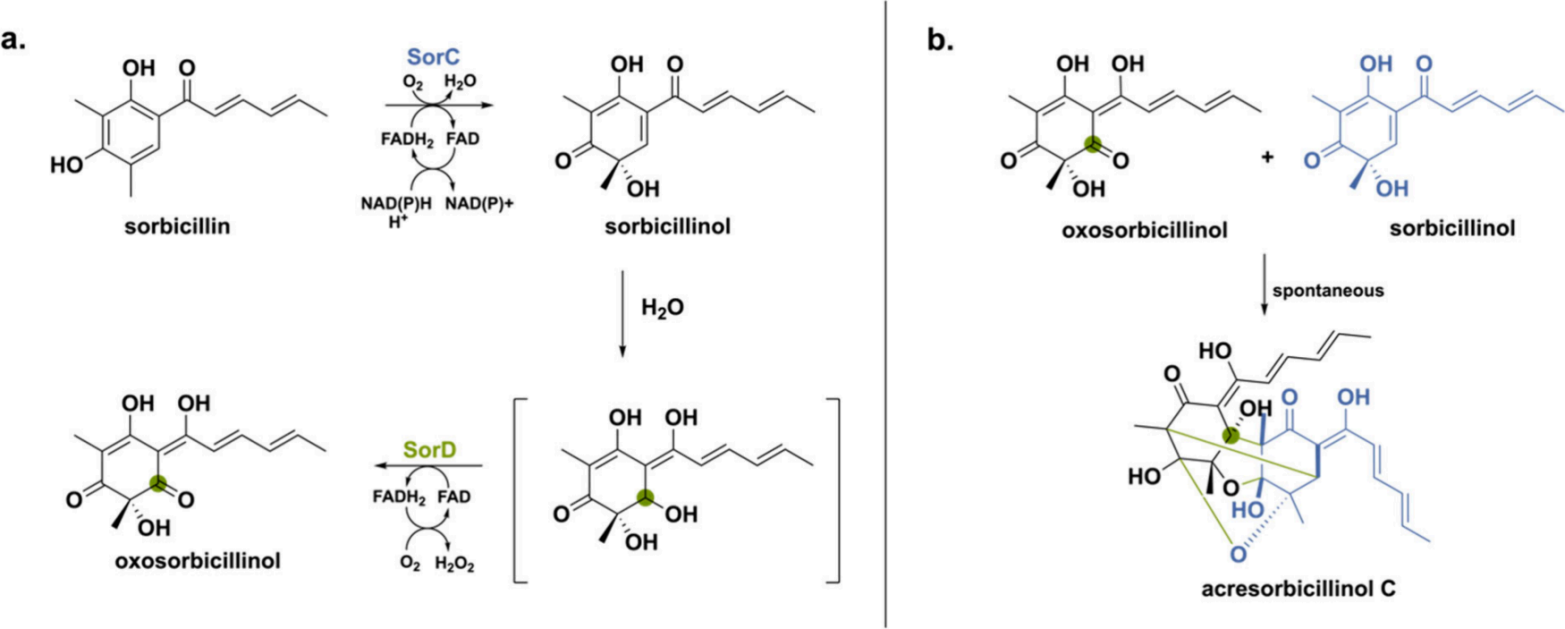
(a) The NAD(P)H-Dependent Flavoprotein Monooxygenase SorC Installs a Hydroxy-Group in Sorbicillin Producing the Reactive Sorbicillinol and (b) the Reactive Sorbicillinol Undergoes a Michael-Addition Reaction with Oxosorbicillinol to Produce the Dimer Acresorbicillinol C After spontaneous hydration, the BBE-like oxidase SorD performs an alcohol oxidation to form oxosorbicillinol.

Another more recent example is a BBE-like oxidase involved in sorbicillinoid biosynthesis.^56^ Sorbicillinoids represent a substantial fungal natural product class encompassing over 100 derivatives characterized by intricate three-dimensional architectures and a wide spectrum of significant pharmacological activities.^57^ Sorbicillinoids are categorized into monomeric, dimeric, trimeric, and hybrid groups based on shared structural features. The biosynthetic pathway toward sorbicillinoids involves the collaboration of a highly reducing iterative polyketide synthase (hrPKS, SorA) and a nonreducing iterative polyketide synthase (nrPKS, SorB) to establish the core backbone of sorbicillin. Subsequently, an NAD(P)H-dependent flavoprotein monooxygenase SorC catalyzes the oxidative dearomatization to yield sorbicillinol. The last enzyme in the biosynthetic pathway is BBE-like oxidase SorD that has been shown to play a crucial role in the polymerization and oxygenation of sorbicillinol.^36,58,59^ Recently, SorD from *Acremonium chrysogenum* was heterologously expressed in *Aspergillus nidulans* to analyze its exact function.^56^ Sorbicillinol undergoes spontaneous hydration, as it is a highly reactive α,β-unsaturated ketone generating the hydrated sorbicillinol intermediate (Scheme 5). Subsequently, SorD oxidizes this intermediate to the corresponding oxosorbicillinol. Oxosorbicillinol can then spontaneously react with sorbicillinol to form the cage-like acresorbicillinol C by a Michael-addition reaction.

**Scheme 6:**
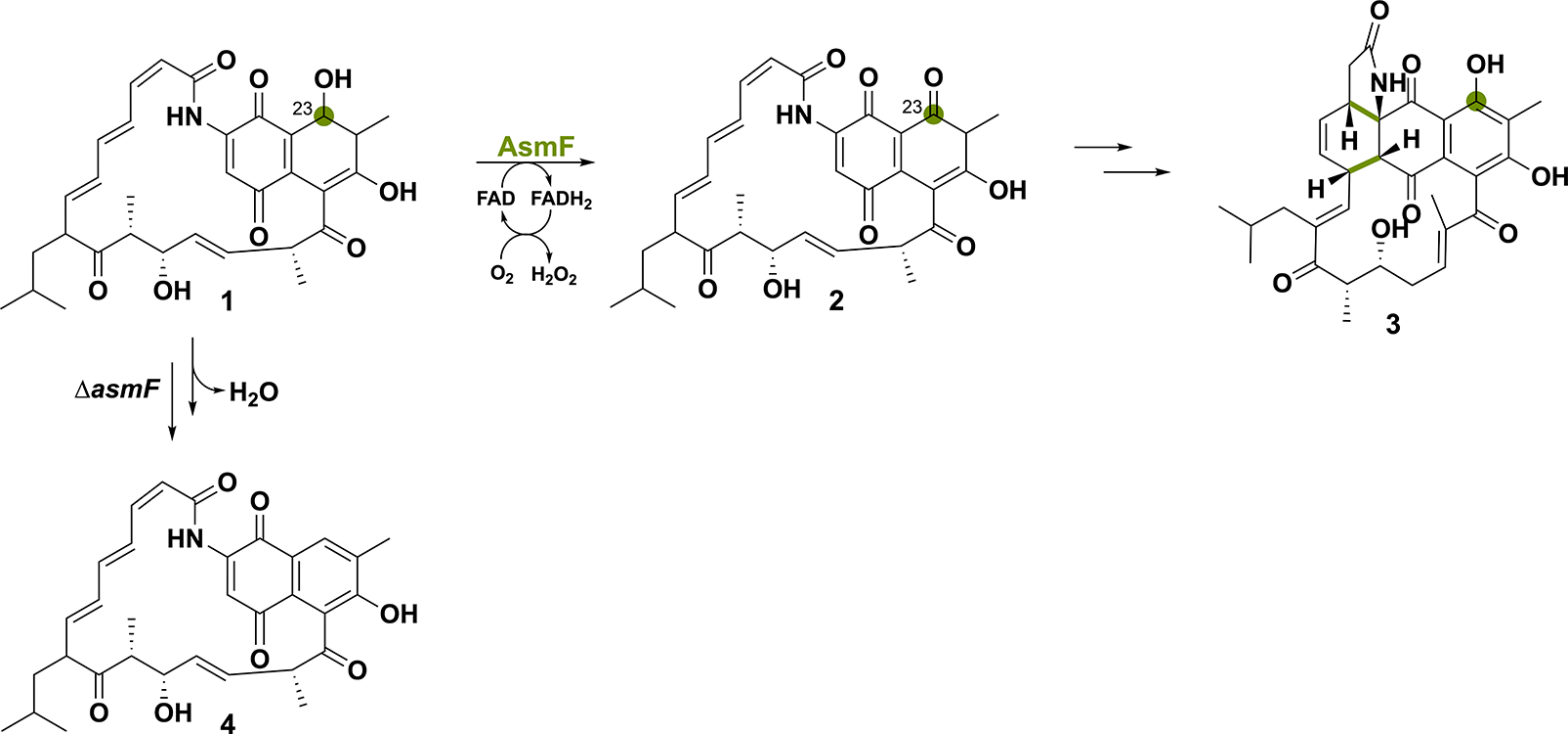
Oxidoreductase AsmF Catalyzes the Alcohol Oxidation to the Corresponding Ketone, Preventing Spontaneous Dehydration and Therefore Limits Ansamycin Derivatization

Ansaseomycins are part of the macrolactam family and exhibit potent biological activities.^60^ The oxidoreductase AsmF was shown to be responsible for the oxidation of C23-OH of the nascent polyketide synthase product **1** to yield ketone compound **2** (Scheme 6). This paves the way, after keto–enol tautomerization, to a Diels–Alder reaction forming compound **3**.^37^ Knocking *asmF* out from the heterologous strain of *Streptomyces seoulensis* A01 led to an increase in novel ansaseomycin derivatives. Indeed, when the hydroxyl group was not modified by AsmF, the substrate could undergo spontaneous dehydration, forming **4**, a common precursor for other deoxy-naphthalenic compounds. This observation implies that nature has developed a specific approach for incorporating the hydroxyl naphthalenic moiety in ansamycin natural products. Ultimately, the deletion of *asmF* might be an effective strategy to enhance the structural diversity of ansamycins.

**Scheme 7:**
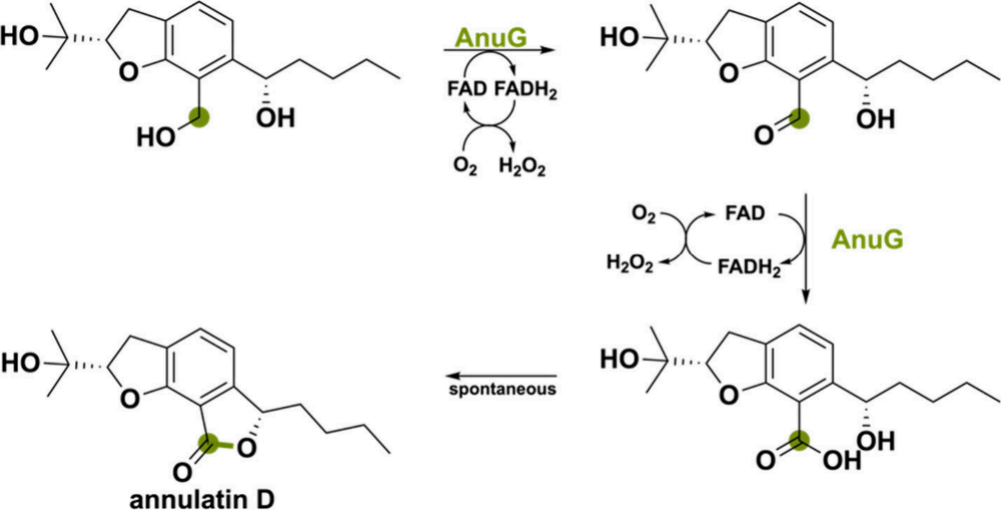
Double-Oxidation Reaction Performed by AnuG to Create the Natural Product Annulatin D

AnuG was identified in the silent biosynthetic gene cluster involved in the oxidative lactonization forming the polyketide annullatin D.^61^ Via overexpression in *A. nidulans*, it was shown to synthesize the five-membered lactone ring via the oxidation of the hydroxyl groups of the alkylated salicylaldehyde precursor (Scheme 7). It is postulated that the hydroxymethyl functional group undergoes a double oxidation to the acid.^61^ The first oxidation creates an aldehyde that, after the addition of water, forms a hydrate that can be oxidized to the acid. This is followed by spontaneous lactonization with the other hydroxyl group. A homologous BGC can be found in *Aspergillus ruber*, *Neurosporin crassa* and *Trichoderma virens*, where the AnuG orthologs correspond to FogF, SrdI and VirF with sequence identities of 27–28%.^62−64^ These oxidases are thought to be involved in the oxidation of the hydroxymethyl group to the aldehyde of a flavoglaucin congener, but they do not lead to the formation of five-membered lactones. This is because the flavoglaucin congener substrate lacks the hydroxyl group needed for intramolecular ring-closure. The cytochrome P450 monooxygenase (CYP) responsible for installing this hydroxyl group is not present in the BGC of *N. crassa*, and in *A. ruber*, the respective CYP hydroxylates the aromatic ring instead of the side chain. In *T. virens*, the CYP has not been characterized.

**Scheme 8:**
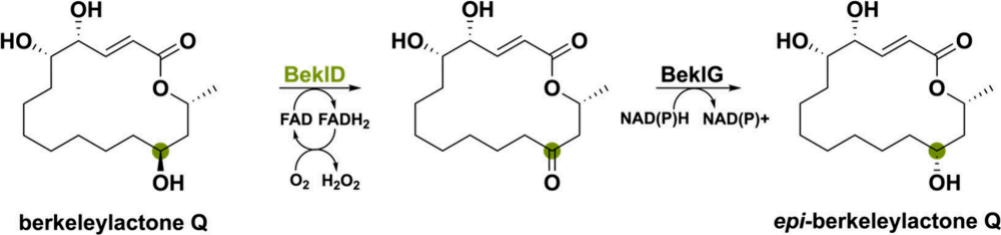
Epimerization of Berkeleylactone Potentially Catalyzed by the Oxidase BeklD and Short-Chain Dehydrogenase/Reductase BeklG

Another notable enzyme is BeklD, a bicovalently-FAD bound flavoprotein that is likely to enable epimerization of berkeleylactone derivatives by performing an alcohol oxidation.^65^ Berkeleylactones are macrocyclic polyketides produced by several *Penicillium* species.^66,67^ Even though it is postulated that a short-chain dehydrogenase/reductase (SDR) termed BeklG is responsible for the complete epimerization, it might instead be a combination of the SDR BeklG and oxidase BeklD (Scheme 8). Characterized homologues of BeklD have been found in other macrolide-producing organisms such as BerkD from *Penicillium egyptiacum* (96% seq id.) and ZEB1 from *Fusarium graminearum* (40% seq id.) where they perform a secondary alcohol oxidation.^66,68^ The ketone functional group could then be reduced into the other epimer by the SDR BeklG, thereby enabling epimerization and causing structural diversification.

**Scheme 9:**
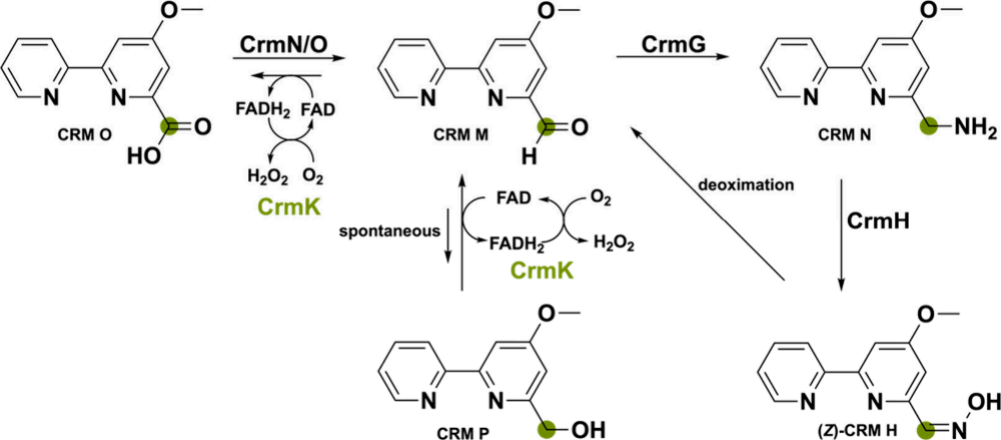
Salvage Pathway for the Secondary Metabolite Caerulomycin A in Which CrmK Can Perform a Double Oxidation to Recycle the Alcohol to the Corresponding Aldehyde CRM M and Acid CRM O

A flavoprotein found in the marine-derived fungus *Actinoalloteichus cyanogriseus* WH1–2216–6 is noteworthy as it does not partake in the biosynthesis of the nonribosomal peptide-polyketide hybrid secondary metabolite caerulomycin A itself.^69,70^ This bicovalently FAD-bound oxidase named CrmK can recycle products back to the main biosynthetic pathway via a double oxidation reaction of an alcohol to a carboxylate via an aldehyde (Scheme 9). The on-pathway carboxylate substrate CRM O is transformed into the corresponding aldehyde intermediate CRM M using the dehydrogenase pair CrmN/CrmO. This aldehyde substrate is then stepwise converted to the *Z*- and *E*-configured aldoxime CRM H by the two-component monooxygenase CrmH. The unstable *Z*-CRM H, however, can spontaneously go back to the aldehyde CRMN M by a deoximation reaction. This would lead to an accumulation of the aldehyde intermediate. Since aldehydes are known for being reactive electrophiles, they are often detoxified by the cell enzymatically or spontaneously. In the cell, the aldehyde CRM M can spontaneously reduce to the corresponding alcohol CRM P in an inadvertent side reaction to potentially avoid buildup of the aldehyde intermediate. However, CrmK can shunt it back to the aldehyde and, after hydrate formation with water, also oxidize it to the carboxylate CRM O although with lower efficiency. Intriguingly, CrmK enables the possibility of a salvage pathway, which is rarely seen in secondary metabolism and perhaps more unassigned genes in BGC could have this function.^71−73^

**Scheme 10:**
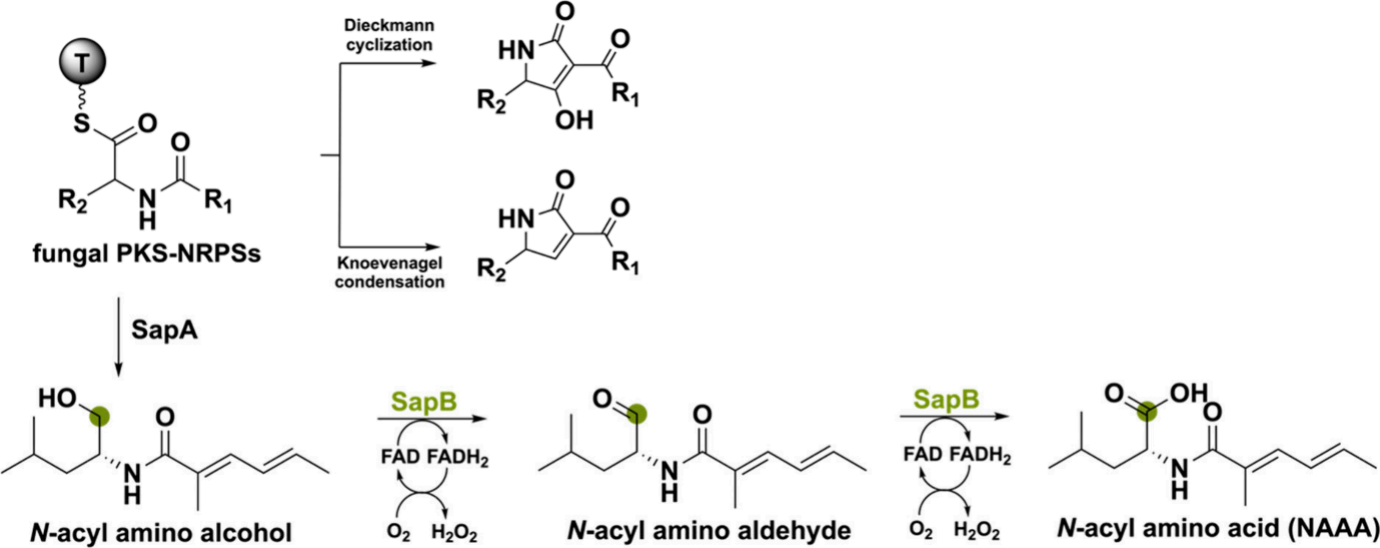
Four-Electron Oxidation Performed by SapB to Form an *N*-Acyl Amino Acid in *Scedosporium apiospermum* F41-1 Fungal PKS-NRPSs have different release mechanisms, and SapA uses a four-electron reduction to release the *N*-acyl amino alcohol from the thiolation domain (T).

Another flavoenzyme capable of a four-electron oxidation is called SapB from the *sap* cluster in *Scedosporium apiospermum* F41–1.^74^ This cluster encodes a PKS-NRPS (*sapA*) and flavin-dependent oxidoreductase (*sapB*) that together assemble a polyketide–amino acid (PKAA) conjugate type *N*-acylated amino acid (NAAA) that inhibits *Arabidopsis* root growth. Generally, the C-terminal domain of a fungal PKS-NRPS is reductive and can only release the amino-acyl adduct intermediate through a Dieckmann cyclization or Knoevenagel condensation making it impossible for a single fungal PKS-NRPS to produce a PKAA conjugate.^75^ Interestingly, SapA, after backbone formation, does not use such a release mechanism. Instead, it forms an *N*-acylated amino alcohol as an intermediate which can be used by the BBE-like oxidase SapB to perform a double oxidation creating the NAAA (Scheme 10). This work shows a new biosynthetic reasoning leading to a novel PKAA conjugate type NAAA enabled by combining a PKS-NRPS with a BBE-like oxidase.

**Scheme 11:**
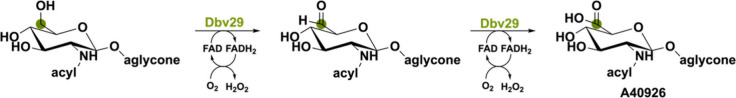
Four-Electron Oxidation Catalyzed by Dbv29 in the Maturation of Glycopeptide A40926

The flavoprotein Dbv29 is the first described FMN-containing bicovalent oxidase.^76^ Dbv29 is involved in the maturation of the glycopeptide A40926, a vancomycin-like glycopeptide, and an important antibiotic obstructing bacterial cell wall synthesis. By catalyzing two consecutive oxidation reactions, Dbv29 transforms the *N*-acyl aminoglucosamine into *N*-acyl aminoglucuronic acid (Scheme 11). Liu et al.^77^ have been able to synthesize new antibiotic analogues for combatting antibacterial resistance by rationally intercepting the aldehyde intermediate and performing a chemoenzymatic reductive amination using different amines.^78^

**Scheme 12:**
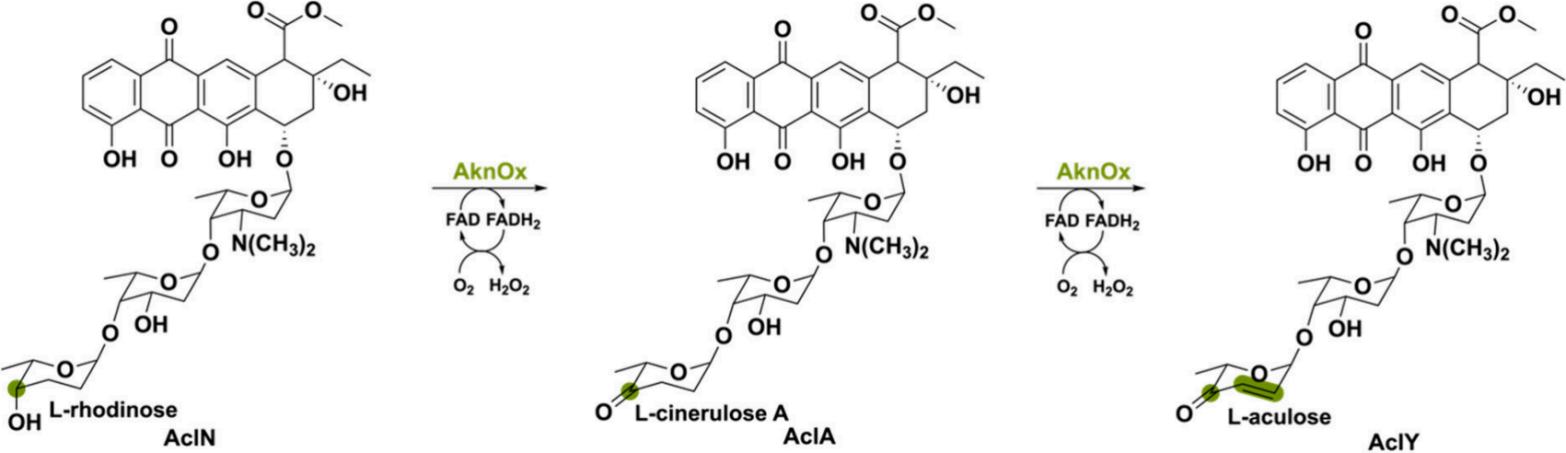
Oxidation of Two Different Functional Groups (−CH–OH and −CH_2_CH_2_) Performed by the Flavoenzyme AknOx Using Two Separate Sets of Active Site Residues

Another unusual flavoenzyme is the aclacinomycin oxidase named AknOx. This bacterial-derived oxidase from *Streptomyces galilaeus* MA144-M1 is also capable of a four-electron oxidation but on two different functional groups: a – CH–OH and – CH_2_–CH_2_ moiety.^79^ Aclacinomycins (Acl) are aromatic polyketides with antibiotic and antitumor activity, hence a compound of interest for disease treatments.^80^ Connected to Acl is a trisaccharide moiety of which the terminal L-rhodinose sugar residue is modified by AknOx (Scheme 12). The next biosynthetic step involves the elimination of two hydrogen atoms in L-cinerulose A to form L-aculose, thereby obtaining AclY. The same active site is utilized for the catalysis of two FAD-dependent steps in the biosynthesis of AclY. Nevertheless, two separate sets of active site residues are used for each reaction, making AknOx a special flavoenzyme.

**Scheme 13:**
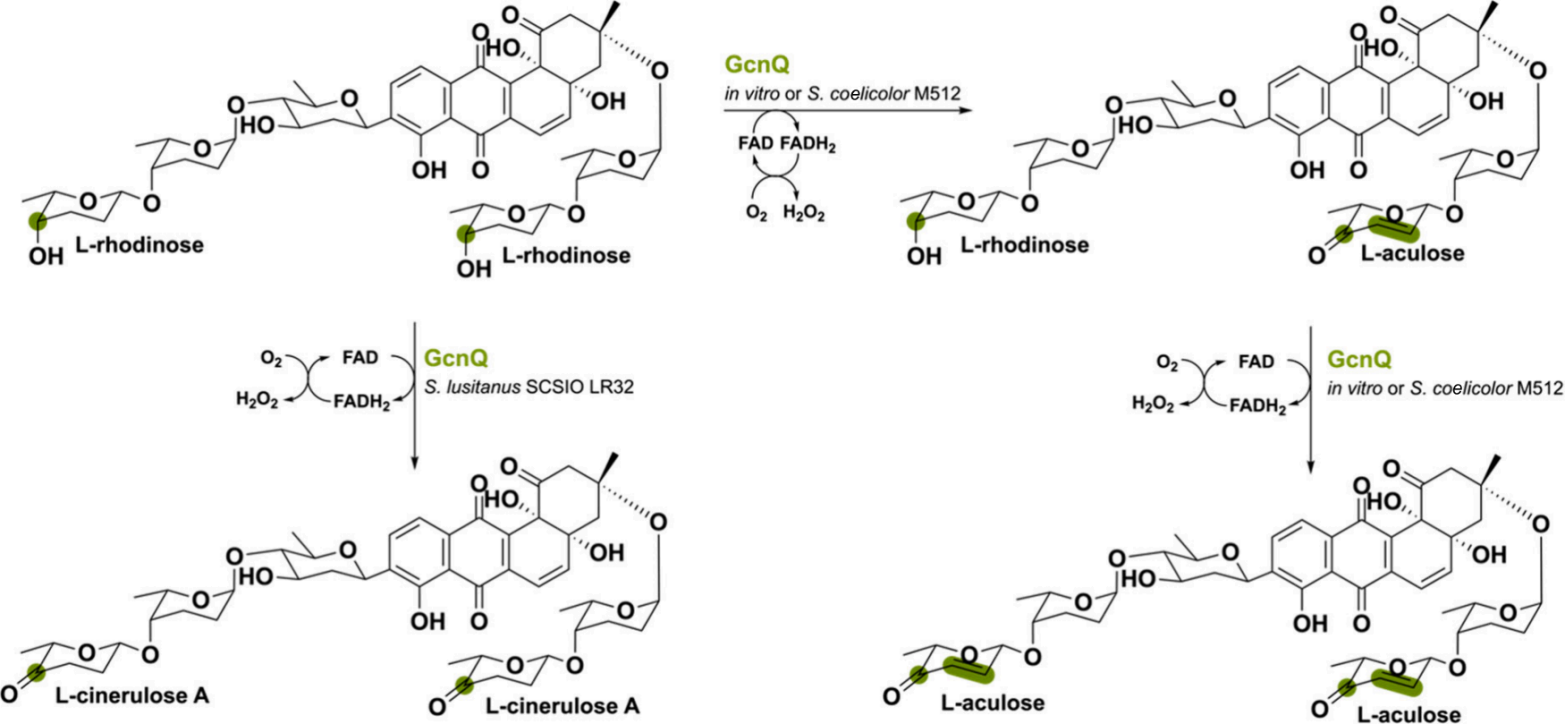
Oxidation Reactions Catalyzed by GcnQ *In Vitro* Producing Different Grincamycin Derivatives Depending on the Host Organism and Reaction Conditions

A similar flavoenzyme named GcnQ (56% seq identity to AknOx) has been found in the grincamycin gene cluster of *Streptomyces lusitanus*.^81^ Grincamycin (GCN) has a tetragomycin skeleton containing a di- and trisaccharide substituent both with a terminal L-rhodinose moiety. Interestingly, *in vitro* this bicovalent FAD-containing GcnQ is able to perform the same double oxidation of the terminal sugar L-rhodinose to L-aculose. It was shown that the oxidation and subsequent desaturation occur in tandem without forming the intermediate L-cinerulose A (Scheme 13), differently to what has been observed in aclacinomycin biosynthesis.^79^ Moreover, GcnQ has divergent roles when it is expressed in different hosts. In *S. lusitanus* SCSIO LR32, GcnQ manages to perform solely the single oxidation reaction of L-rhodinose to L-cinerulose A. However, when being heterologously expressed in *S. coelicolor* M512 it is able to transform the L-rhodinose units to L-aculose. This exemplifies the diverse activities that a single berberine bridgelike enzyme can exhibit in natural product biosynthesis.

**Scheme 14:**
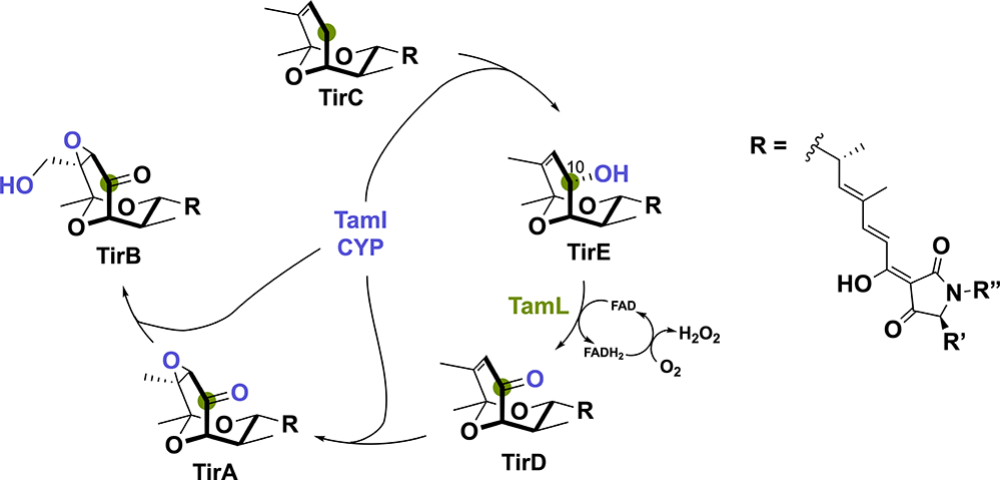
Biosynthetic Pathway for the Natural Product Tirandamycin Where the CYP TamI Initiates the Cascade via Hydroxylation of TirC to TirE The flavin-dependent oxidase TamL converts TirE to the corresponding ketone TirD, which is again a substrate for TamI.

TamL is a flavoprotein derived from *Streptomyces* sp. 307–9 and involved in the biosynthesis of the antibiotic tirandamycin.^82^ This flavin-dependent oxidase displays intriguing enzymatic interplay with a cytochrome P450 monooxygenase (CYP) named TamI in effectively tailoring tirandamycin.^83^ The oxidative reaction occurs in a defined order, where TamI first performs hydroxylation of substrate TirC to TirE (Scheme 14). Thereafter, TamL must oxidize this C10 hydroxyl group to the corresponding ketone TirD. TirD is then again used a substrate for TamI converting it to TirA via epoxidation and subsequently to TirB by a final hydroxylation reaction. This iterative substrate exchange between TamL and TamI establishes a distinctive tailoring route wherein a CYP performs multiple oxidations in tandem with another biosynthetic enzyme. Together, these oxidative alterations within the tirandamycin pathway play a substantial role in enhancing its antibiotic potency toward inhibiting bacterial ribonucleic acid polymerase by increasing reactivity.^84,85^

**Scheme 15:**
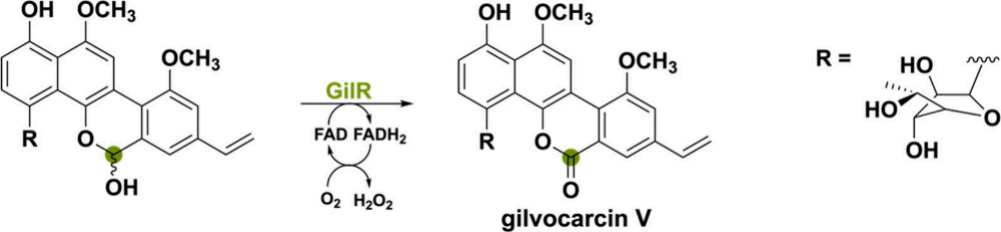
GilR Catalyzing the Oxidation of a Hemiacetal to the Lactone Gilvocarcin V

Another bacterial-derived BBE-like oxidase is GilR from *Streptomyces griseoflavus* Gö3592.^86^ This unusual lactone-forming oxidoreductase catalyzes the final step in the biosynthetic pathway toward the polyketide-derived gilvocarcin V. Gilvocarcin-type compounds show distinct anticancer activity through a light-mediated [2 + 2]-cycloaddition adduct with the side chain of DNA.^87,88^ GilR is responsible for oxidizing the hemiacetal to the lactone, thereby forming gilvocarcin V (Scheme 15). This lactone moiety is of crucial importance for the antibiotic’s stability and potency.^89^

### *ortho*-Quinone Methide Intermediate

A classical mechanism observed for a wide variety of BBE-like oxidases is the deprotonation of a phenolic moiety with subsequent or concomitant hydride transfer at a distant carbon atom. The aromaticity of phenolic hydroxyl groups enables the delocalization of electrons and the transfer of a hydride from a distant carbon atom to the N5-atom of FAD. This deprotonation-assisted distant hydride transfer reaction can go via an ο-QM intermediate and multiple BBE-like oxidases have been hypothesized to employ this oxidation mechanism.^46^

**Scheme 16:**
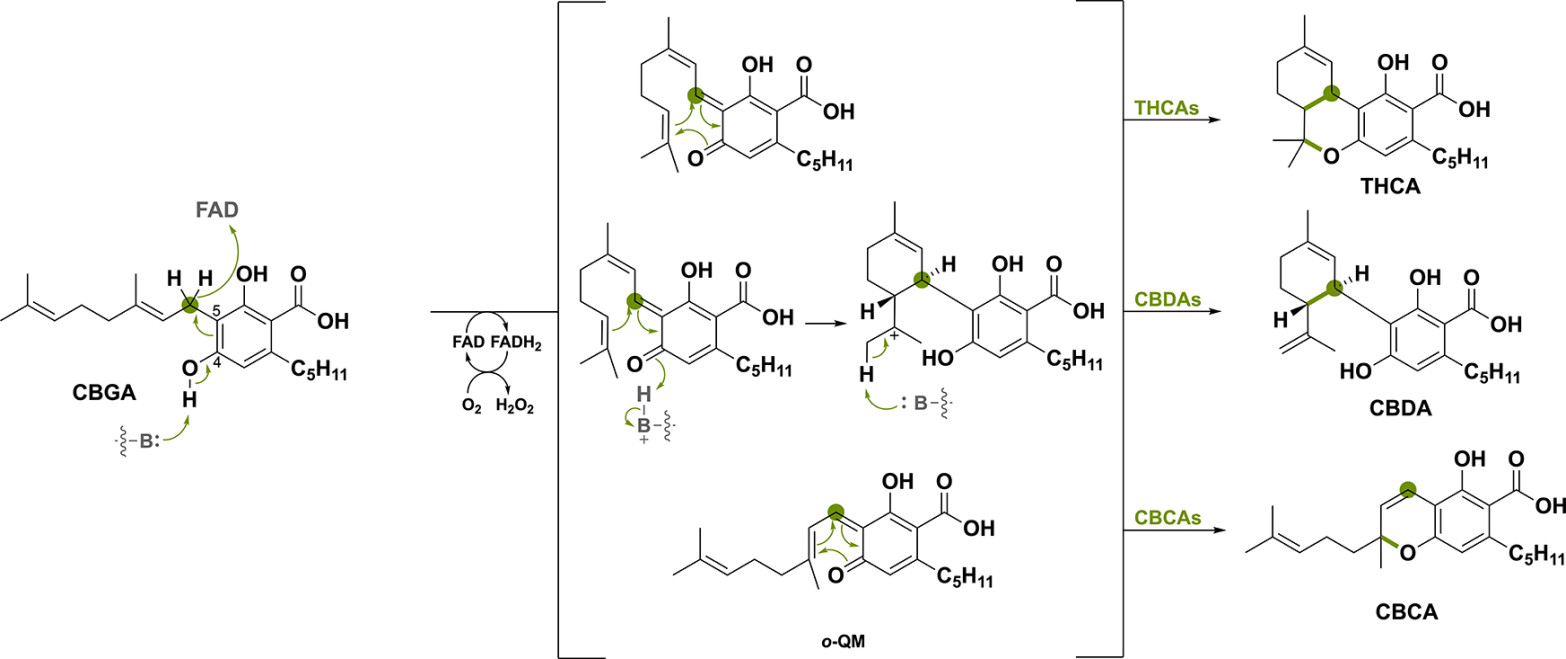
Deprotonation-Assisted Distant Hydride Transfer of Cannabigerolic Acid (CBGA) to Form an ο-QM Intermediate Three BBE-like oxidases use this intermediate to generate three different cannabinoids (THCA, CBDA, and CBCA) via specific intramolecular cyclization reactions.

The first example encompasses three BBE-like oxidases that are involved in the biosynthesis of the primary cannabinoids found in *Cannabis sativa*,^90−92^ namely tetrahydrocannabinolic acid synthase (THCAs), cannabidiolic acid synthase (CBDAs) and cannabichromenic acid synthase (CBCAs).^45,93^ These three BBE-like oxidases act on the same precursor, cannabigerolic acid (CBGA), and share an oxidative carbon–carbon bond formation. Purdy et al.^46^ proposed that the reaction is initiated by deprotonation of the C4 phenolic hydroxy group by a crucial tyrosine acting as a catalytic base (Scheme 16). This allows for subsequent or simultaneous transfer of the hydride to the oxidized FAD cofactor, generating the ο-QM intermediate. The following intramolecular cyclization can go through different mechanisms and regioselectivities, generating the three different cannabinoids THCA, CBDA and CBCA.

**Scheme 17:**
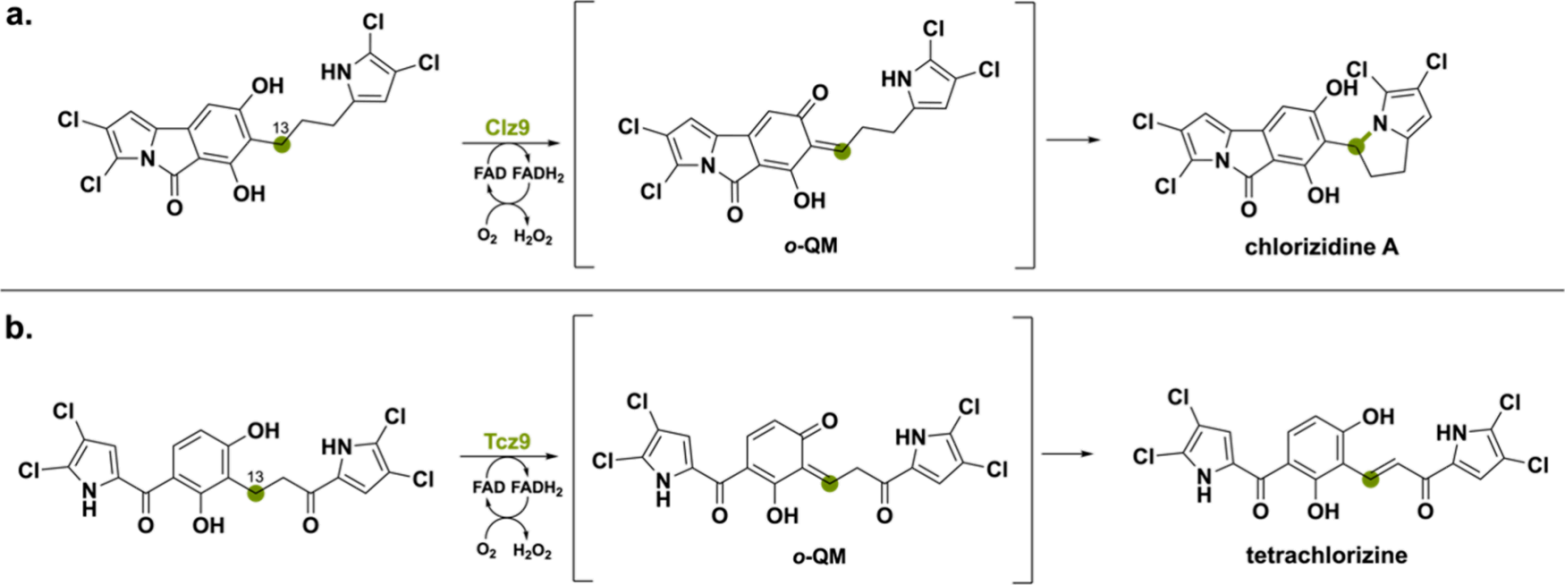
(a) The Oxidase Clz9 Catalyzes the Oxidation-Mediated Intramolecular Cyclization via an ο-QM Intermediate Forming Chlorizidine A and (b) the Oxidase Tcz9 Catalyzes the Benzylic Dehydrogenation via an ο-QM Intermediate Forming Tetrachlorizine

Another BBE-like oxidase that is involved in oxidative cyclization via an ο-QM intermediate is Clz9 found in the BGC of *Streptomyces* sp. CNH-287 producing chlorizidine A.^94^ This natural compound contains a pyrrolo-isoindolone ring, which is a new and unique structure in the field of natural products.^95^ Deprotonation of the phenolic hydroxyl moiety promotes hydride transfer from the benzylic C13 to FAD, forming an ο-QM intermediate. The enone is then attacked by the pyrrole’s nitrogen creating chlorizidine A (Scheme 17a). A similar enzyme has been identified from *Actinomycete* strain AJS-327 where a homologous (53% seq. id.) FAD-dependent oxidoreductase was found and named Tcz9.^38^ The oxidation reaction is again proposed to include an ο-QM intermediate but instead of a nucleophilic attack of pyrrole’s nitrogen, it forms a dichloropyrrole-containing compound featuring an α,β-unsaturated ketone (Scheme 17b).

**Scheme 18:**
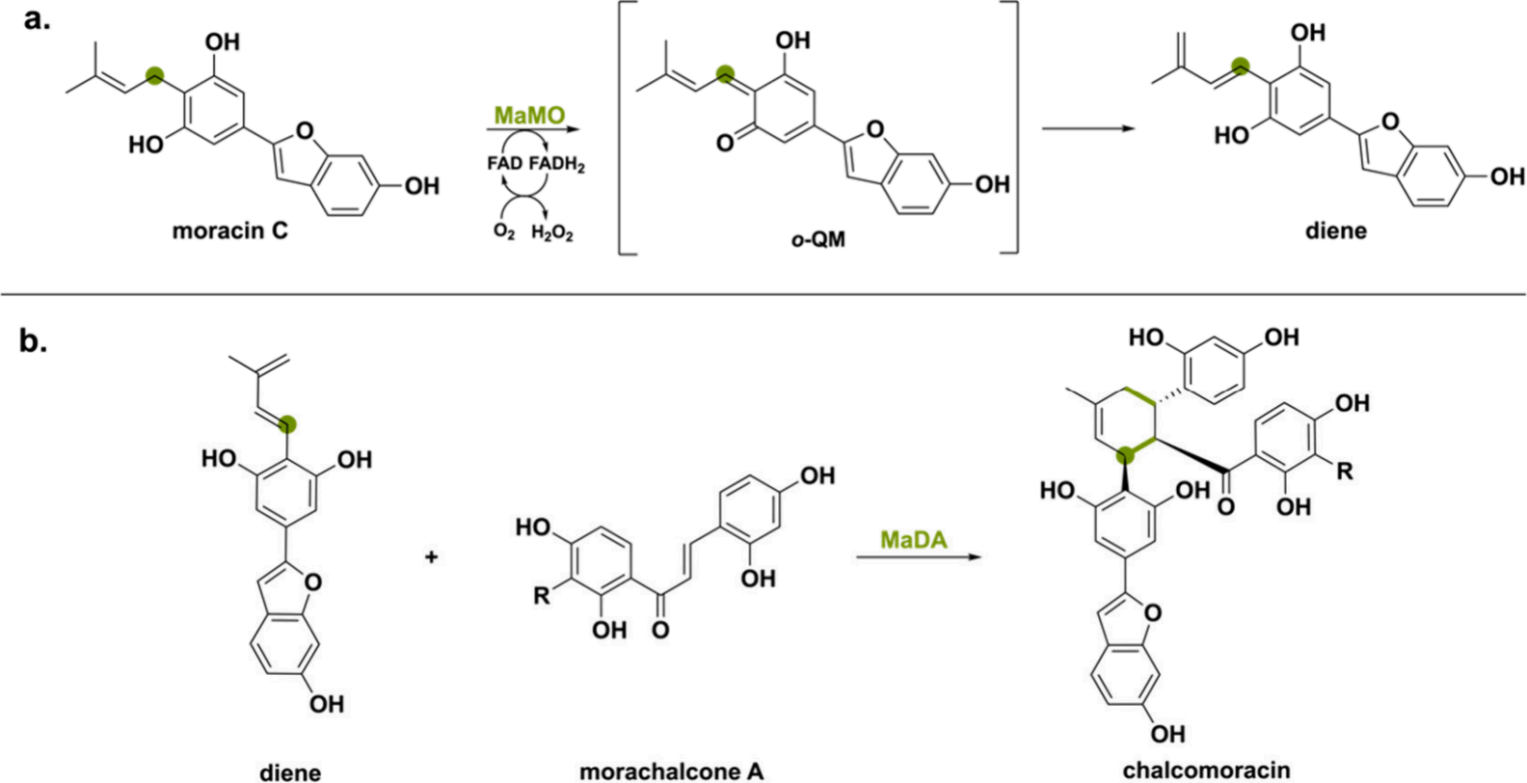
(a) The BBE-like Oxidase MaMO Catalyzes the Oxidation of the Isoprenyl Moiety of Moracin C via an ο-QM Intermediate and (b) the Second BBE-like Oxidase MaDA Catalyzes the [4 + 2] Cyclization of the Formed Diene with Morachalcone A to Generate the Natural Product chalcomoracin

ο-QM intermediate can also be found in the secondary metabolic pathway of mulberry plants (*Morus alba*).^33^ Once a plant gets infected by a fungus, it produces chalcomoracin as a mean to protect the leaves via fungal germination suppression.^96^ This flavonoid is formed through an enzymatic Diels–Alder reaction between a diene (the isoprenyl portion of an isoprenylphenol) and a dienophile (double bond of morachalcone A). Interestingly, there are two BBE-like enzymes involved in this specific biosynthetic pathway and they show high (50%) sequence identities with THCAs, CBDAs and CBCAs.^45^ First, the BBE-like moracin C oxidase (MaMO) oxidizes the isoprenyl moiety of moracin C into the diene (Scheme 18a). The authors suggest a similar ο-QM intermediate in which deprotonation of the aromatic hydroxy group of moracin C leads to hydride transfer and subsequent diene formation via tautomerization. Next, the second BBE-like enzyme named Diels–Alderase (MaDA) enables [4 + 2] cyclization of the formed diene with the dienophile morachalcone A, representing the first known stand-alone intermolecular Diels–Alderase (Scheme 18b). MaDA will be discussed in more detail in the later section “Other Mechanisms”.

**Scheme 19:**
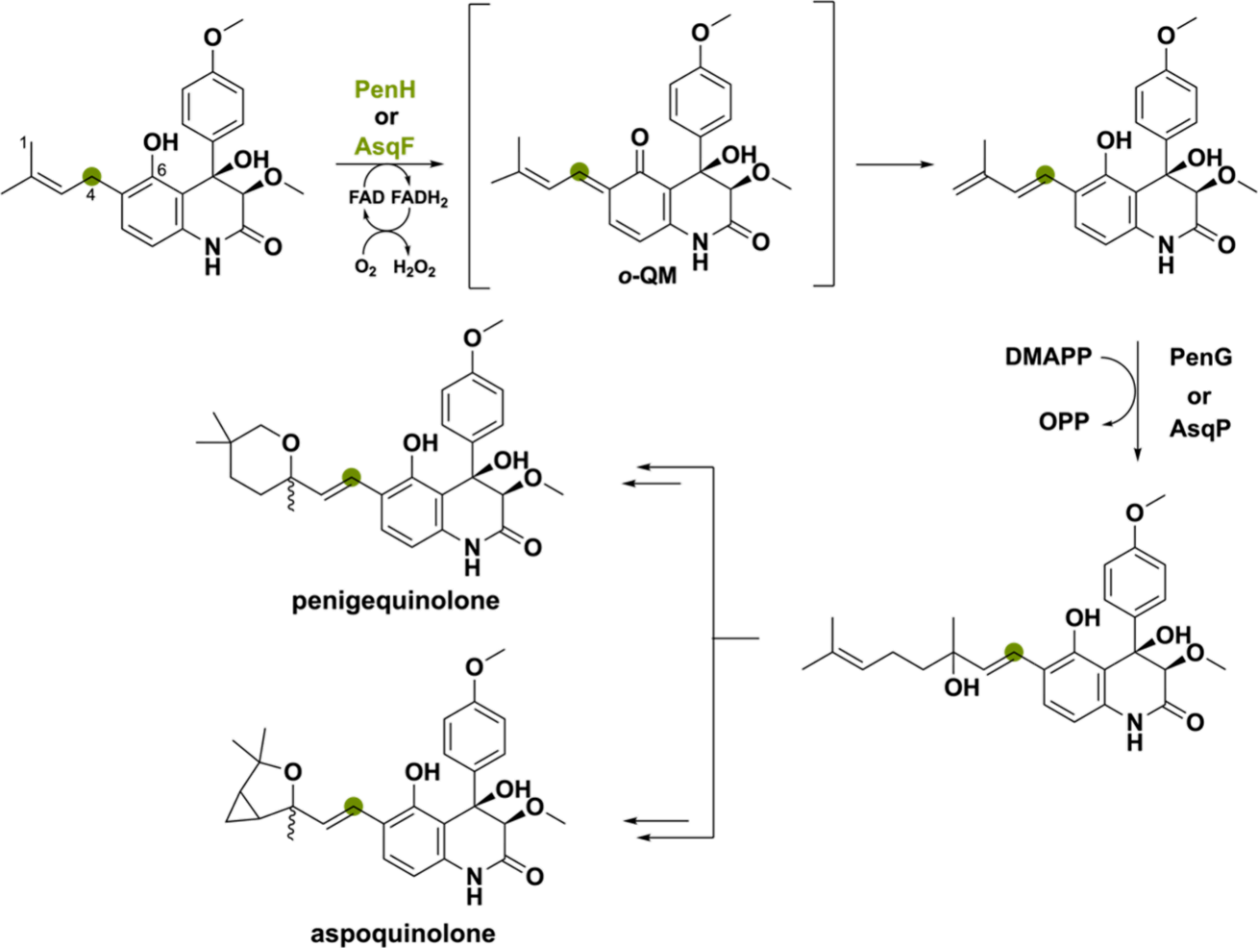
Oxidation of the Isoprenyl Side Chain Proposed to Go via an ο-QM Intermediate Catalyzed by PenH in the Biosynthesis of Penigequinolone or AsqF in the Biosynthesis of Aspoquinolone

Two closely related BBE-like oxidases, PenH and AsqF, perform an atypical oxidation-mediated extension of the prenyl side chain in the biosynthesis of the penigequinolone and aspoquinolone alkaloids of *Penicillium thymicola* and *A. nidulans*.^97−99^ A base-catalyzed reaction was proposed in which the C4 proton is removed and the C1 hydride is transferred to the N5 atom of the FAD cofactor.^40^ However, based on recent research of ο-QM intermediates, it might be more plausible that proton abstraction of the C6 phenolic alcohol promotes hydride transfer (Scheme 19). Both mechanisms produce an electron-rich diene intermediate able to undergo alkylation by dimethylallyl diphosphate as catalyzed by the prenyltransferase PenG or AsqP. Thereafter, the chain-extended product undergoes branching leading to penigequinolone or aspoquinolone.

**Scheme 20:**
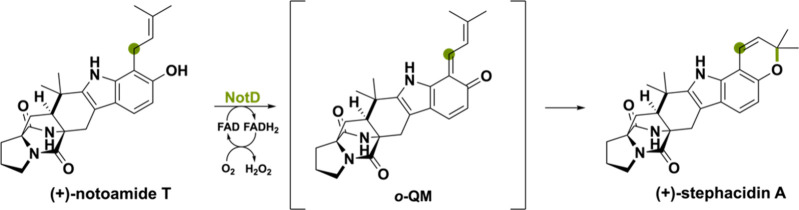
Oxidative Pyran Ring Formation Thought to Be Catalyzed via an ο-QM Intermediate The reaction displayed is the conversion of (+)-notoamide T to (+)-stephacidin A by the BBE-like oxidase notD.

Three BBE-like oxidases are involved in production of the cytotoxic paraherquamide A, (−)-notoamideA and (+)-notoamide A.^100,101^ So far, research has not elucidated the exact roles of the corresponding flavoenzymes PhqH in *Penicillium fellutanum*, NotD in *Aspergillus* sp. MF297–2 and NotD’ in *Aspergillus versicolor* NRRL35600. However, they are thought to be responsible for the oxidative pyran ring formation reaction (Scheme 20). It is plausible that the oxidation of the prenyl side chain goes via an ο-QM intermediate leading to a [4 + 2] cycloaddition reaction forming the corresponding pyran moiety. Below, the reaction is displayed for NotD from *Aspergillus* sp. MF297–2.^102^

**Scheme 21:**
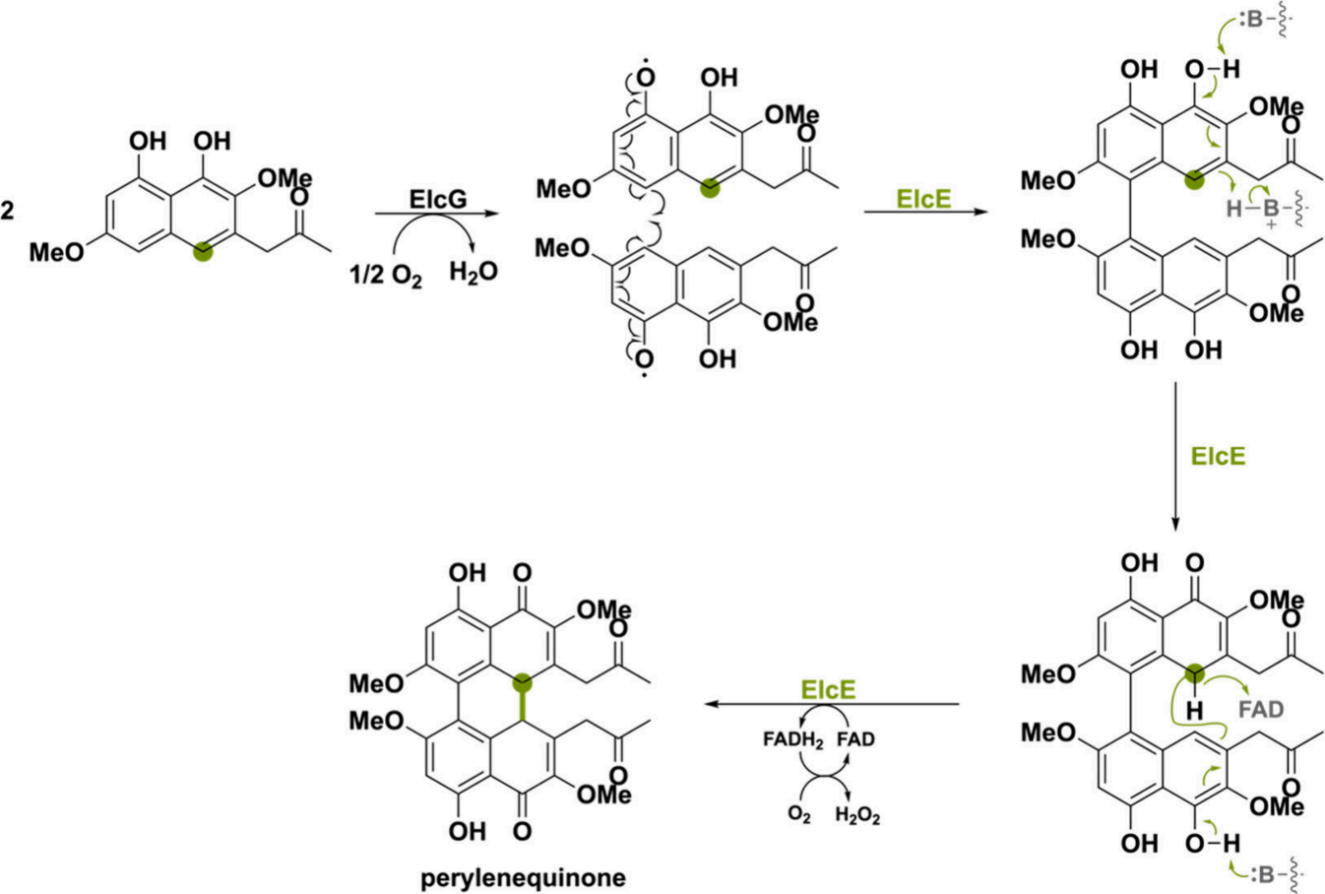
Double Coupling of Two Napthol Derivatives Thought to Be Catalyzed by the Laccase-like Multicopper Oxidase ElcG and Flavin-Dependent Oxidase ElcE

Another peculiar BBE-like oxidase is ElcE involved in the biosynthesis of the aromatic polyketide perylenequinone elsinochrome A found in *Parastagonospora nodorum*. ElcE was found to be responsible for the coupling of two nahpthol derivatives, requiring a laccase-like multicopper oxidase ElcG.^103^ The same was observed in *Cercospora nicotianae*, where the ElcG homologue, CTB12, was deleted thereby completely abolishing cercosporin production.^104^ By heterologous biosynthesis, it was demonstrated that both ElcE and ElcG are needed for the double coupling step, yielding the pentacyclic perylenequinone (Scheme 21). As laccases are known for catalyzing radical coupling reactions,^3^ it was suggested that ElcG catalyzes the first coupling via a phenol radical coupling mechanism. Thereafter, ElcE would catalyze the second carbon–carbon bond formation initiated by phenolic hydroxyl deprotonation and a concomitant or subsequent distant hydride transfer. Scheme 21 shows the suggested pathway proposed by Hu et al.^103^ However, the exact mechanism has yet to be elucidated.

### Amine and Amide Oxidation

**Scheme 22:**
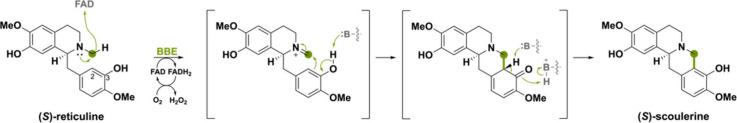
Proposed Reaction Mechanism of the Plant-Derived Berberine Bridge Enzyme (BBE) Forming (*S*)-Scoulerine via Formation of the Berberine Bridge

Many BBE-like enzymes are effective amine oxidases that generate transient imines or iminium cations that can undergo derivatization. The name-bearer of the BBE-like subfamily catalyzes this type of oxidation reaction (Scheme 2).^23^ A concerted mechanism was proposed where deprotonation of the C3-phenol, hydride transfer from the *N*-methyl group to the FAD and ring closure occur in a single step.^27^ However, a later-performed study on the solvent and substrate deuterium kinetic isotope effect suggested that an earlier proposed stepwise mechanism would be more likely.^29,105^ In the stepwise mechanism, a hydride is transferred from the *N*-methyl group forming a methylene iminium ion intermediate (Scheme 22). Thereafter, the active-site base Glu417 deprotonates C3-phenol, making the C2 carbon more nucleophilic and able to attack the *N*-methylene moiety, forming the berberine bridge.

**Scheme 23:**
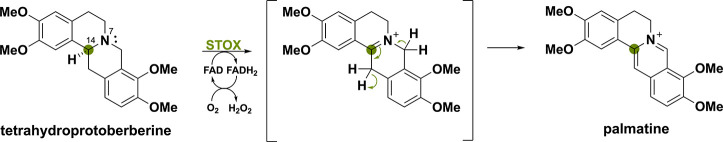
Amine Oxidase Tetrahydroprotoberberine Oxidase (STOX) Catalyzing the Four-Electron Oxidation Reaction Transforming Tetrahydroprotoberberine into Palmatine

A similar amine oxidase involved in benzylisoquinoline alkaloid biosynthesis is the (*S*)-tetrahydroprotoberberine oxidase (STOX) from *Berberis wilsoniae*.^106,107^ STOX can oxidize C–N bonds of the protoberberine alkaloid tetrahydroprotoberberine to produce palmatine (Scheme 23).^108^ Moreover, STOX has high sequence similarity to the BBE name-bearer from *E. californica* with 38%. Unsurprisingly, BBE itself also displays STOX activity although with lower efficiency.^109^ A four-electron oxidation reaction has to take place to synthesize palmatine and the initial oxidation forming the iminium ion is thought to proceed enzymatically and the second oxidation to proceed spontaneously.^106^ Mechanistic investigations concluded that the iminium ion was formed between C14 and N7, creating an unstable intermediate that can undergo spontaneous oxidation in the presence of O_2_.

**Scheme 24:**
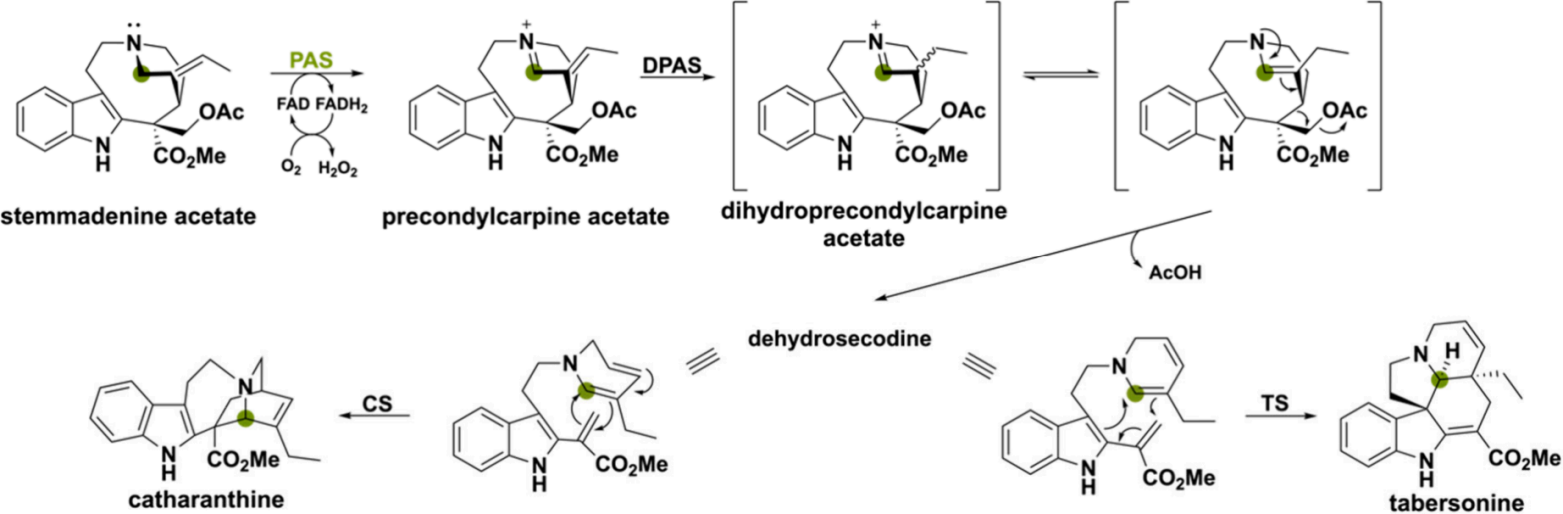
Amine Oxidation Catalyzed by the BBE-like Enzyme PAS, Enabling Subsequent Transformations to Produce the Secondary Metabolites Tabersonine and Catharanthine

Another example involves the monoterpenoid indole alkaloids catharanthine and tabersonine, produced by the plant *Catharanthus roseus*. These alkaloids are intermediates in the biosynthesis of the potent anticancer drugs vincristine and vinblastine and a BBE-like amine oxidase is involved in the synthesis of the scaffolds.^110^ This BBE-like enzyme was named precondylocarpine acetate synthase (PAS) and together with dihydroprecondylocarpine synthase (DPAS), tabersonine synthase (TS) or catharanthine synthase (CS) act in a concerted manner to produce tabersonine and catharanthine (Scheme 24).^34^*In vitro* analysis showed that PAS oxidizes the C–N bond of stemmadenine acetate producing the iminium intermediate precondylocarpine acetate, which is subsequently reduced by DPAS and undergoes [4 + 2] cyclization by TS or CS. Interestingly, PAS lacks the His and Cys residues necessary for the FAD cofactor’s covalent attachment. Homologues of PAS, sharing amino acid identities ranging from 68% to 74%, have been identified in other Apocynaceae plant species, suggesting their potential involvement in the assembly of similar monoterpene indole alkaloids through a conserved mechanism.^110^

**Scheme 25:**
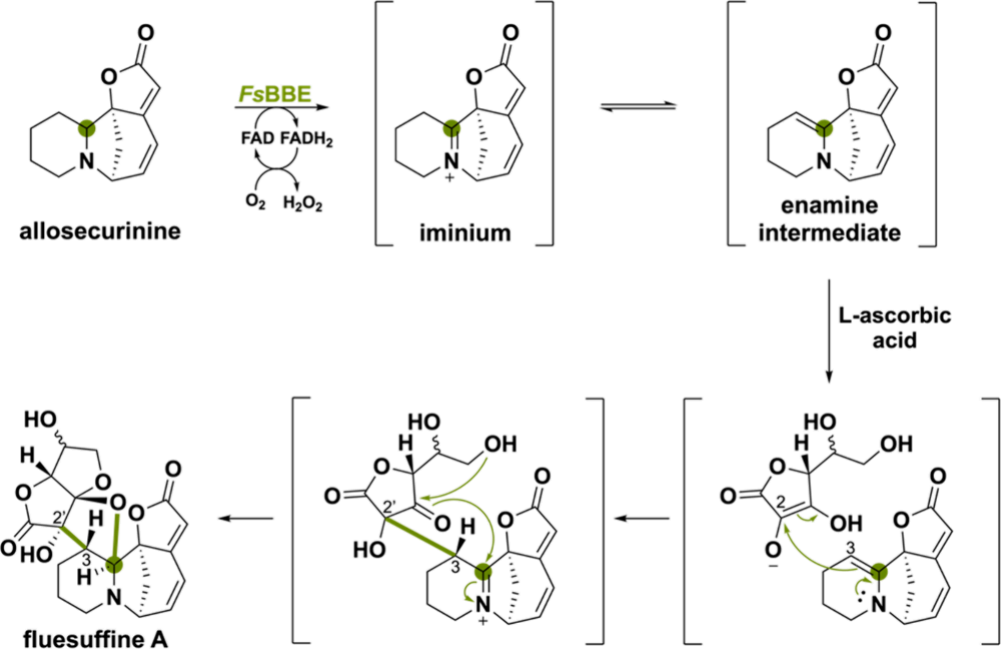
Intermolecular Enamine-Type Addition Reaction Catalyzed by the Plant-Derived *Fs*BBE via an Initial Amine Oxidation

A peculiar BBE-like enzyme named *Fs*BBE has been found in the biosynthetic pathway of plant-derived *Securinega* alkaloids that enables intermolecular enamine-type addition reactions.^111^ The substrate allosecurinine is oxidized by *Fs*BBE to form the iminium ion and tautomerization generates the enamine intermediate (Scheme 25). The C3 atom can then act as a nucleophile and attack the C2 of l-ascorbic acid (or its oxidative product dehydroascorbic acid). This enamine-type addition reaction enables a spontaneous cascade reaction of ketal formation and cyclization to fluesuffine A. Previously, it was proposed that many C2 or C3-funtionalized *Securinega* alkaloids were produced via a key enamine intermediate which acts as a branching point for further derivatization.^112,113^ In this study, *Fs*BBE has shown to be a relevant gene for the biosynthesis of enamine intermediate, which opens up a way to generate novel C2 and C3-functionalized *Securinega* alkaloids.

**Scheme 26:**
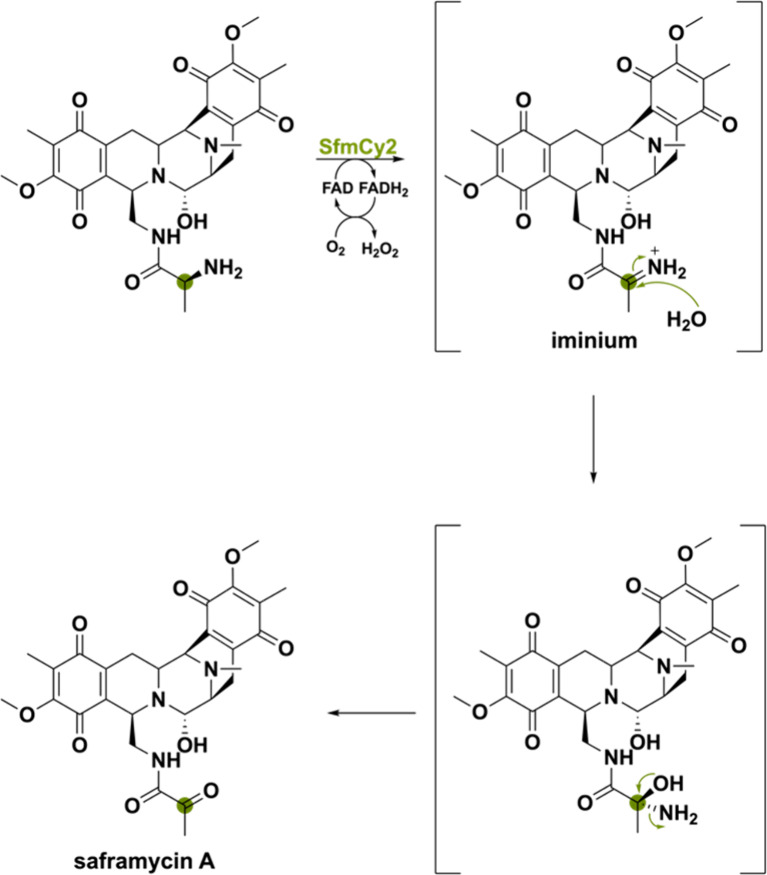
Oxidative Deamination by SfmCy2 Allowing for Prodrug-Maturation of Saframycin A in *Streptomyces lavendulae*

The flavoprotein SfmCy2 makes use of a transient iminium ion to accomplish the oxidative deamination forming the natural product saframycin A.^114^ SfmCy2 is expressed extracellularly by bearing a Tat signal peptide and allows for prodrug-maturation of saframycin A in *Streptomyces lavendulae* by catalyzing a final deamination reaction. This reaction proceeds through dehydrogenation of the amino group to form an imine intermediate (Scheme 26). The resulting imine is attacked by water to form the hemiaminal, which after NH_3_ release forms the ketone. The reaction was also performed in H_2_^18^O and afforded an enzymatic product with a molecular weight increase of 2 Da as compared to the reaction performed in H_2_^16^O. This validates that the ketone group is originating from water. This study uncovers another unique activity that BBE-like enzymes exhibit, namely, oxidative deamination.

**Scheme 27:**
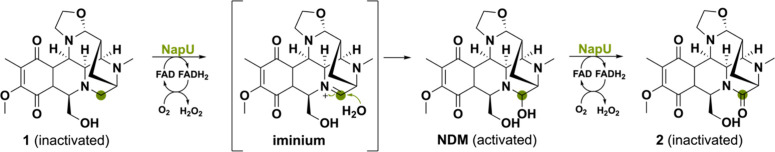
Oxidative Activation and Overoxidative Deactivation of Napthyridinomycin (NDM) by the Flavoprotein NapU in *Streptomyces lusitanus* NRRL 8034

Another secreted BBE-like protein from *Streptomyces* bearing a Tat signal peptide is named NapU and performs an oxidative activation and overoxidative inactivation of the matured prodrug naphthyridinomycin (NDM).^115^ The first reaction that NapU catalyzes is the hydroxylation of the pharmacophore inactivated compound **1** to form the bioactive NDM (Scheme 27). When the reaction mixture was incubated for a longer period of time, another product **2** was formed with a decrease in molecular weight of 2 Da that did not show antibacterial activity. The first hydroxylation reaction is proposed to go through the reactive iminium intermediate. H_2_O may act as the nucleophile, potentially activated by two Tyr active site residues, to attack the imine and install the hydroxy-group in NDM. It was illustrated that NapU can also mediate the subsequent two-electron oxidation, yielding the corresponding inactivated ketone-derivative. Hence, the flavoprotein NapU is involved in the activation of a matured prodrug, but can also avoid self-toxicity via overoxidation of NDM as self-defense strategy.

Although there is a considerable number of identified FAD-dependent amine oxidases,^116^ amide oxidases are not commonly known in literature. They are predicted to be involved in the biosynthesis of certain fungal-derived natural products such as pyranonigrin and pyranterreones.^117,118^ The first study that fully characterized a BBE-like oxidase performing an unprecedented amide oxidation is FmqD, which operates in the biosynthesis of two cytotoxic peptidyl alkaloids, fumiquinazoline C (FQC) and D (FQD).^47,119^ These metabolites are a key feature of the pathogenic fungus *A. fumigatus* and have received considerable attention due to their complex biochemistry.^120^ Thanks to co-ordination with sporulation-specific transcription factors, FmqD is secreted to the cell wall and directs its product to the fungal spores.^120^

**Scheme 28:**
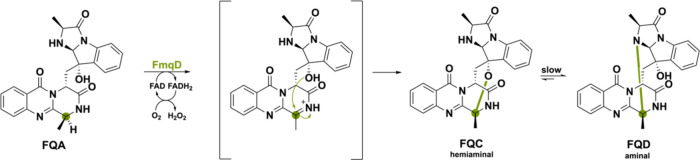
Amide Oxidation of FQA by Oxidase FmqD Allowing Intramolecular Cyclization Producing the Natural Products FQC and after Slow Equilibration FQD

FmqD primes its so-called substrate fumiquinazoline A (FQA) for C–O and C–N intramolecular cyclization. Specifically, amide oxidation leads to a transient imine, which is captured by the −OH group of the imidiazoindolone side chain, producing the spirohemiaminal FQC (Scheme 28).^47^ Slow equilibration ultimately forms the aminal FQD as a more thermodynamically stable product. The absence of the lone-pair stabilization for the amide might seem to make hydride abstraction of the α-carbon challenging, but it is speculated that the extended delocalized system of the FQ scaffold manages to stabilize the cation to allow for hydride abstraction. This example illustrates the delicate chemistry required for amide oxidation, leading to regioselective product cyclization.

### Monoterpene Indole Alkaloids

**Scheme 29:**
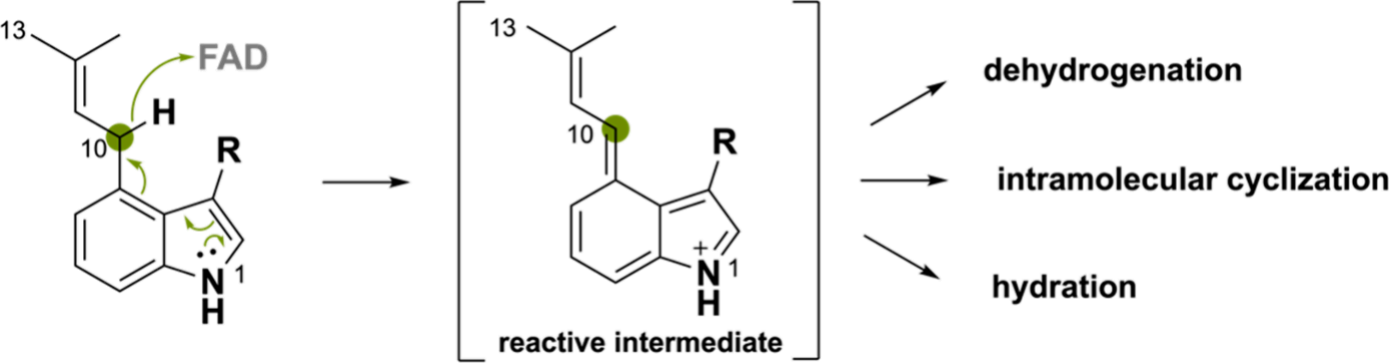
Proposed Oxidation Mechanism for Monoterpene Indole Alkaloids, Where Hydride Transfer at the C10 Center Is Enabled through Electron Pair Delocalization at the N1 Atom Forming the Reactive Iminium Ion Intermediate

A few BBE-like enzymes oxidize an isoprenyl moiety, typically conjugated to an indole-derived scaffold and also called monoterpene indole alkaloids. The mechanism by which this occurs has not yet been understood, and some propose the hydride transfer to occur at the benzylic C10 atom assisted via abstraction of the C13 hydrogen or vice versa. Taking into account the other BBE-like oxidation mechanisms discussed so far, we propose that the hydride transfer is enabled by the electron donating effect of the N1 atom through the indole ring to the C10 atom (Scheme 29). This ultimately allows the hydride to be transferred to the N5 atom of FAD. Such a mechanism would be similar to the one of the ο-QM intermediate.^46^ The obtained reactive iminium ion intermediate can undergo a multitude of derivatizations including intramolecular cyclizations, dehydrogenations, and hydration reactions.

**Scheme 30:**
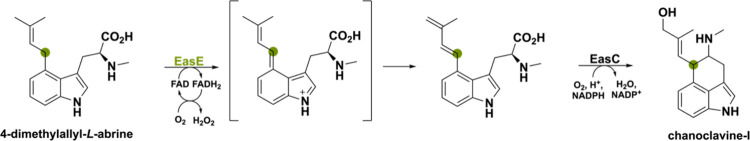
Isoprenyl Oxidation of the Ergot Alkaloid 4-Dimethylallyl-l-abrine by the BBE-like Oxidase EasE, Enabling Subsequent Oxidative Cyclization by Catalase EasC

The first example can be found for ergot alkaloids, which are prenylated indole alkaloids produced by several filamentous fungi and have wide applications in therapeutics due to their similarity with monoamine neurotransmitters such as dopamine and adrenaline.^121−123^ A BBE-like oxidase involved in the biosynthesis of ergot alkaloid *D*-lysergic acid is EasE, a bicovalent flavoenzyme from the parasitic fungus *Claviceps purpurea*. Together with a catalase partner enzyme EasC, EasE is involved in the oxidative cyclization of 4-dimethylallyl-l-abrine (4DMA) to chanoclavine-I (Scheme 30).^124^ These two enzymes are essential for the central carbon ring in the tetracyclic ergoline core structure. Until recently, it remained an enigma how this central C ring was formed.^39^ Essentially, EasE performs a dehydrogenation reaction after which the O_2_-dependent catalase EasC can complete the cyclization via a radical addition mechanism.

**Scheme 31:**
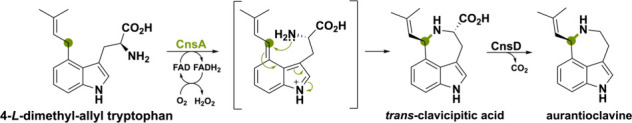
CnsA Catalyzing the Oxidation of the Isoprenyl Moiety with Subsequent Intramolecular Attachment Forming *trans*-Clavicipitic Acid

The FAD-dependent oxidoreductase CnsA and putative catalase CnsD from *Penicillium expansum* share homology to EasE and catalase partner EasC (51 and 59% seq. id.).^48,125,126^ These enzymes operate on 4-l-dimethyl-allyl tryptophan that is oxidized at the isoprenoid unit. However, subsequent cyclization is not caused by a radical addition mechanism but rather by an intramolecular attack of the α-NH_2_ group (Scheme 31). This leads to *trans*-clavicipitic acid, which forms aurantioclavine after decarboxylation by the putative catalase CnsD.

Another type of meroterpenoids is the indole diterpenoids synthesized by various fungi. This meroterpenoid class lacks the polyketide scaffold and instead possesses an indole ring as nonterpenoid part.^127^ Indole diterpenes are generally synthesized via a common hexacyclic molecule named paspaline.^128^ This natural product class obtains variability through branching out using different biosynthetic gene clusters.^129,130^ Here, we will give an overview of three different FAD-oxidoreductases present in the biosynthetic pathways producing the three indole diterpene natural products shearinines, penitrems, and nodulisporic acids.

**Scheme 32:**
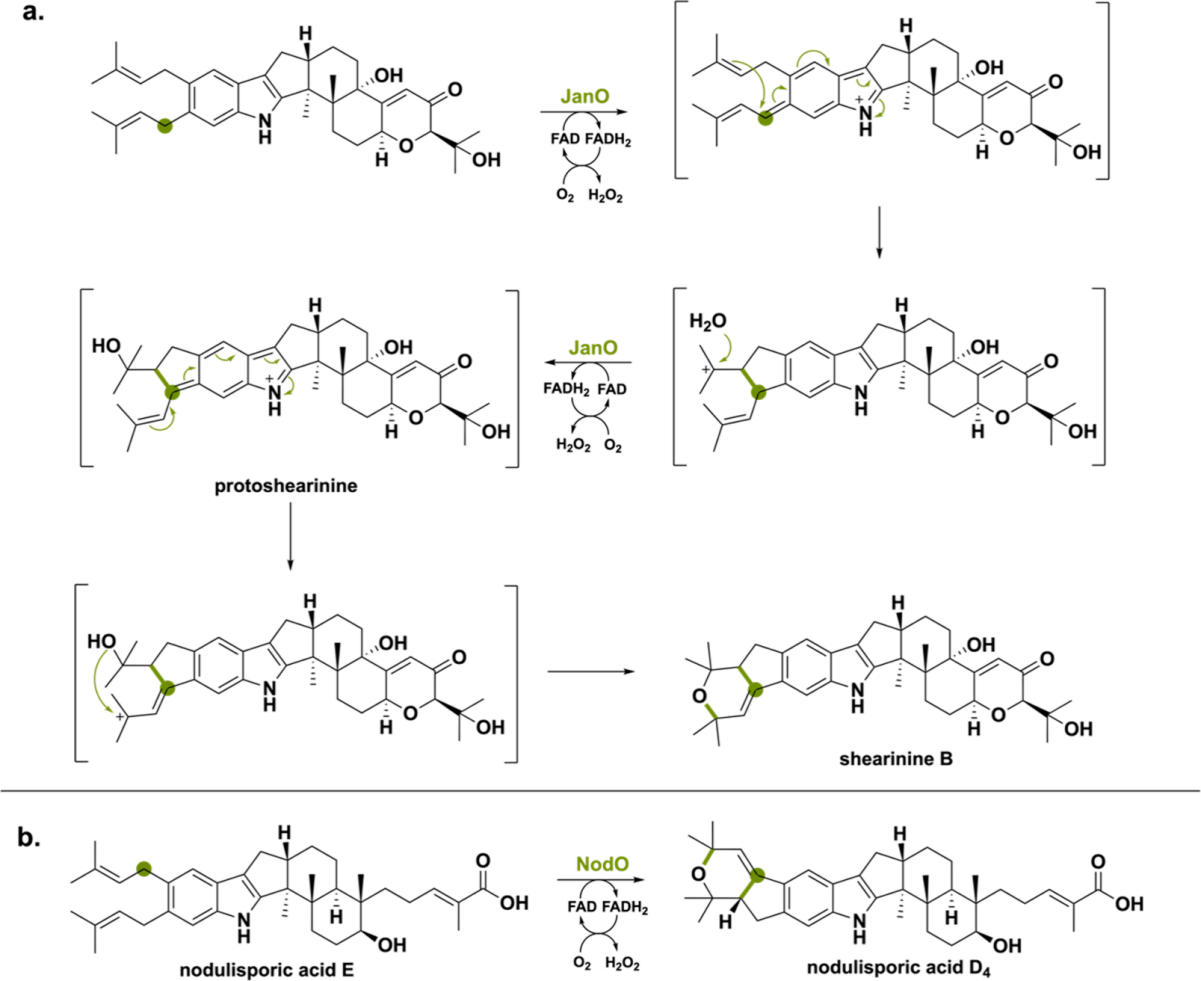
(a) Hypothesized Reaction Mechanism of JanO Allowing for the Double Oxidation of the Isoprenyl Side-Group Thereby Forming the Secondary Metabolite Shearinine B and (b) Oxidative Cyclization Reaction of NodO Producing Nodulisporic Acid D_4_

JanO from *Penicillium janthinellum* and NodO from *Hypoxylon pulicicidum* (previously *Nodulisporium* sp.) perform an intriguing prenylation oxidation and cyclization to form a bicyclic system (Scheme 32).^131−133^ JanO was analyzed *in vitro* and yielded a single product, suggesting that cyclization goes rapidly without intermediate product formation. Incubating the reaction with 30% H_2_O^18^ clarified that the O_2_ atom of the cyclic ether ring was originating from water.^132^ Thus, JanO catalyzes a cascade of reactions: oxidation of the isoprenyl group, hydration, another oxidation, and cyclization yielding the final shearinine B product. Recently, the ortholog of JanO named NodO (52.1% seq id.) was confirmed to be responsible for the oxidative cyclization forming nodulisporic acid D_4_.^131^ Most likely the nodulisporic acid D_4_ synthesis goes via a similar cyclization pathway.^134,135^

**Scheme 33:**
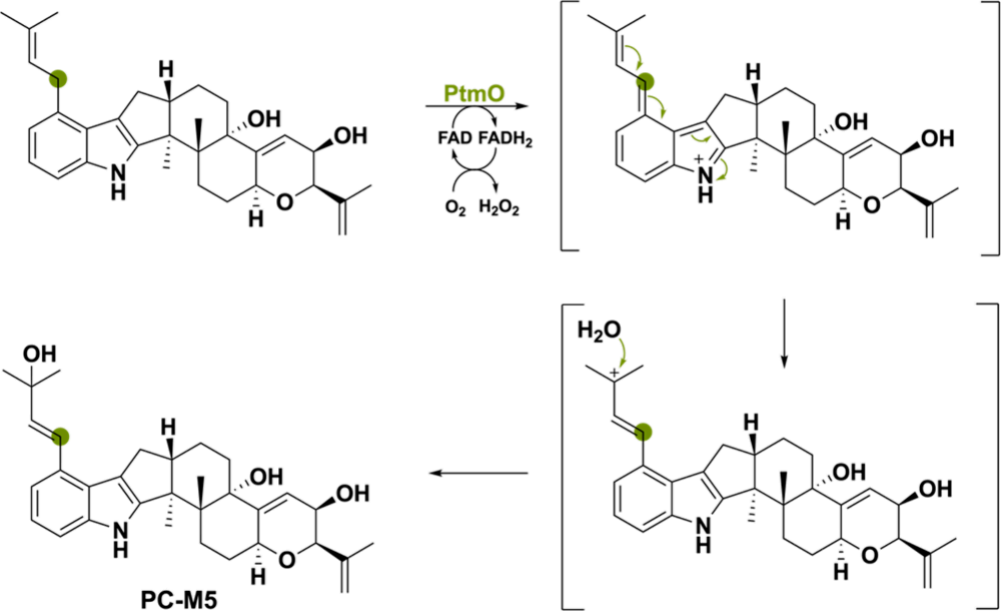
Isoprenyl Oxidation by PtmO Allowing for Water Incorporation and Formation of Compound PC-M5, Subsequently Used as a Substrate for the Formation of Penitrem Derivatives

The third oxidoreductase PtmO from *Penicillium simplicissimum*(136) catalyzes a similar reaction in that its substrate’s isoprenyl group is oxidized, enabling the incorporation of a hydroxyl group coming from water (Scheme 33). The diversity of these reaction illustrates how indole diterpenoids can be used for generating a diverse set of molecules.^130^

### Other Mechanisms

All of the reactions described so far all have an overarching mechanism in which a hydride is transferred to the N5 atom of the FAD cofactor. Nevertheless, examples of BBE-like oxidoreductases can be found that do not employ the FAD cofactor for catalysis. Moreover, there are examples in which the role of the FAD cofactor is still unclear.

The first example is the Diels–Alderase named MaDA,^33,137^ already briefly mentioned during the biosynthesis of morachalcone A in mulberry plants (Scheme 18b). After oxidation of the isoprenyl moiety of moracin C by the BBE-like oxidase MaMO, MaDA enables a [4 + 2] cycloaddition reaction making it the first stand-alone intermolecular Diels–Alderase. Multiple controls were performed to understand the role of the FAD cofactor in MaDa, including mutagenesis of the His116 residue that is critical for covalent FAD attachment and cofactor reduction using sodium dithionite. This mutation rendered MaDA almost inactive, thereby exemplifying that the (oxidized) FAD cofactor is necessary for the *endo* [4 + 2] cycloaddition. Moreover, homologous Diels–Alderases were discovered that could catalyze the same intermolecular Diels–Alder reaction with *exo*-selectivity.^138^ Mutagenesis studies demonstrated key residues involved in activating the dienophile through hydrogen bonding interactions and positioning the diene via π–π interactions for *endo*-selectivity. While for opposite selectivity a crucial arginine residue forms a cation−π interaction with the dienophile, which lowers the free energy barrier for the *exo*-pathway and thereby regulates selectivity. The evolutionary origin of MaDA and MaMO was also investigated and suggested their Diels–Alderase and oxidative dehydrogenation activity to have originated from a gene duplication and subsequent neofunctionalization of an oxidocyclase.^139^ Nevertheless, the exact function of the FAD cofactor in MaDA remains ambiguous.

**Scheme 34:**
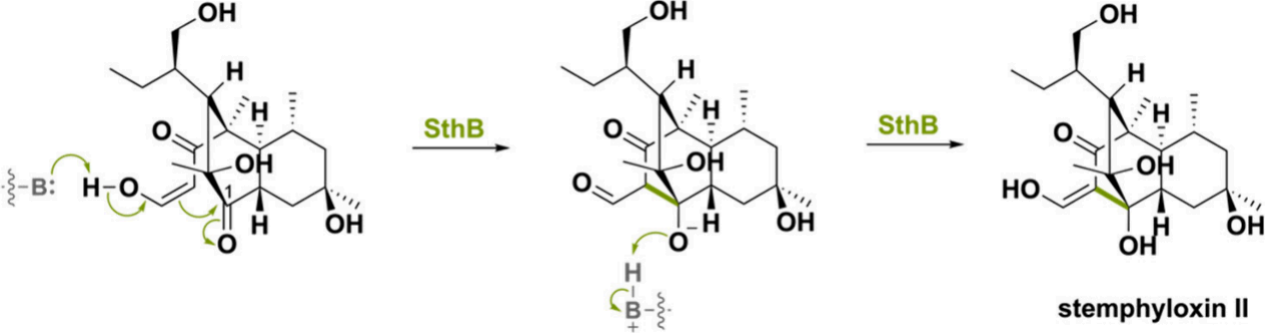
Stereoselective Aldol Reaction Synthesizing Stemphyloxin II is Hypothesized to Go via a Nonoxidative Base-Catalyzed Aldol Reaction in SthB

Another example is the enzyme SthB, involved in the biosynthesis of the phytotoxic polyketide stemphyloxin II.^140^ A highly similar (74% seq. id.) enzyme has been found in betaenone B and C-producing *Phoma betae*.^141^ The striking aspect about SthB is that it catalyzes a stereoselective aldol reaction to form a bridged tricyclo[6.2.2.0^2,7^]dodecane scaffold (Scheme 34). The intramolecular aldol reaction is hypothesized to proceed via the standard base-catalyzed aldol reaction that is nonoxidative. SthB allows for a proton abstraction thereby producing the nucleophilic enolate that attacks the C1-carbonyl on the decalin ring, forming stemphyloxin II. No hydride is being transferred to the N5 atom of the FAD and the role (if any) of the cofactor in SthB has remained unexplored, pending additional biochemical characterization.^142^

**Scheme 35:**
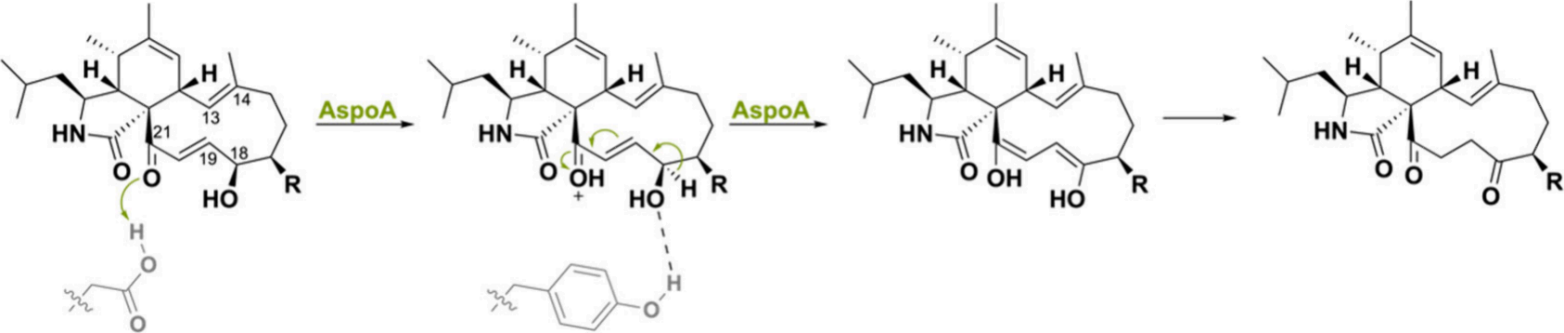
AspoA Acting as a General Acid Biocatalyst Performing a Double Bond Isomerization Reaction

Another peculiar BBE-like oxidase is called AspoA, which is involved in the biosynthesis of cytochalasans, fungal polyketide-nonribosomal peptides with antibiotic properties (PK-NRPs).^143,144^ Interestingly, this flavoenzyme from *Aspergillus flavipes* does not perform a dehydrogenation reaction but acts as general acid biocatalyst to catalyze double bond isomerization (Scheme 35). Addition of FAD did not increase activity, and deletion of His158, which covalently tethers the cofactor, did not decrease activity. A site-directed mutagenesis study was performed to identify the crucial residues and showed that Glu538 was highly conserved as mutagenesis caused a halt in activity. Isotope labeling studies confirmed that the double bond isomerization proceeds via protonation of the C21 carbonyl group, a hydride shift, and a keto–enol tautomerization. AspoA homologues such as ffsJ (85% seq. id.) from *A. flavipes*(145) and PhmC (60% seq. id.) from *P. nodorum*(146) might potentially perform similar reactions as they both harbor the conserved glutamate residue.

**Scheme 36:**
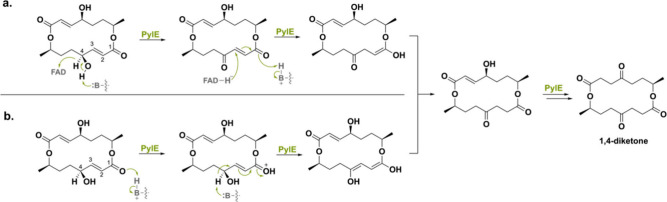
(a) First Proposed Mechanistic Route Used by PylE to Produce the 1,4-Diketone Product and (b) the Second Proposed Route of PylE Using an Acid–Base Residue for the Synthesis of the 1,4-Diketone Product

A distinctive flavoenzyme named PylE was recently found in the biosynthetic gene cluster of *Setosphaeria* sp. SCSIO41009, responsible for the synthesis of pyrenophorol dilactones.^147^ Similar to AspoA (30% seq id), this flavoprotein also catalyzes an isomerization reaction.^143^ In this case, PylE catalyzes the isomerization of the 4-alcohol-2,3-unsaturated moiety of pyrenophorol, producing a 1,4-diketone. Two mechanisms were hypothesized for the isomerization reaction (Scheme 36). In the first mechanism, it was proposed that oxidation of the C4 hydroxyl occurs first, after which a hydride is transferred from FAD to C3 to create the enol intermediate. Tautomerization then leads to the first ketone product. Another isomerization round would then yield the final 1,4-diketone product. The second mechanism goes via initial protonation of the C1 carbonyl, after which the C4 proton is abstracted to form the enol intermediate. Tautomerization and subsequent isomerization of this intermediate create the 1,4-diketone product. Mutagenesis studies proved acid–base residue Glu526 to be a crucial residue for catalytic activity. With the help of docking, the authors showed this residue to be in the vicinity of the C1 carbonyl group and therefore could function as a general acid and base in route b. In contrast to AspoA, mutating the conserved His153 necessary for covalent tethering of the FAD cofactor abolished PylE’s activity. This would suggest its involvement in isomerase activity, but a complete mechanistic study is yet to be performed.

**Scheme 37:**
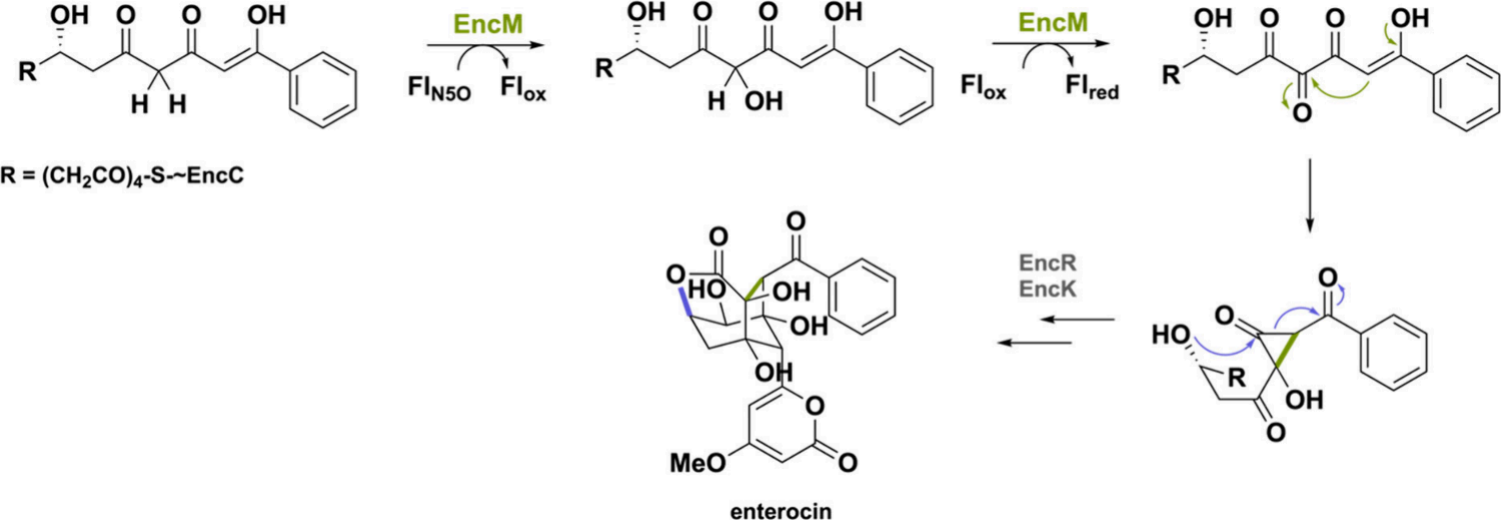
Oxygenation Reaction of EncM Using the Flavin-N5-Oxide Species Allowing the Synthesis of the Natural Product Enterocin

For a long time, oxygenation reactions were commonly thought to be catalyzed by FAD-dependent monooxygenases using the Fl_4aOOH_ species.^9^ Nevertheless, in 2013, the BBE-like enzyme named EncM was shown to feature a different oxygenating species termed as the flavin-N5-oxide (Fl_N5O_).^148,149^ This oxygenase is part of a BGC in *Streptomyces maritimus*, forming enterocin, an unusual polyketide antibiotic. The Fl_N5O_ species triggers a Favorskii rearrangement by simultaneous hydroxylation and dehydrogenation (Scheme 37), reminiscent of the postredox cyclization reactions such as Sol5.^35,55^ So far, EncM has been the only enzyme employing the flavin-N5-oxide intermediate for catalysis within the BBE-like subfamily of oxidoreductases. Nevertheless, this exception sparked the investigation on the versatility of the N5 position and expanded the repertoire of known flavin catalyzed reactions.^150^

## Conclusion

The BBE-like oxidases from the vanillyl-alcohol oxidase/*p*-cresol methyl hydroxylase flavoprotein family are fascinating enzymes exhibiting extraordinary chemical activities. The ability to transfer a hydride atom to the FAD cofactor enables complex substrate modifications and rearrangements, including intramolecular cyclizations, Diels–Alder reactions, Michael additions, and hydroxylations. This leads to a structurally and functionally diverse number of natural products in bacteria, plants, and fungi. A mechanistically recurrent theme of most of these enzymes is the ability to elicit molecular skeleton rearrangements triggered by an initial oxidation step through hydride transfer to the FAD. However, there are also cases in which the exact involvement of the FAD cofactor still remains unknown or in which the FAD cofactor utilizes a different oxygenating species. There is a plethora of uncharacterized BBE-like enzymes, and genomic context is needed to predict their functions. Understanding the natural mechanisms that are employed to generate the incredible diversity of natural products is a topic of great interest in biochemistry for its fundamental implications in the field. Hence, the study and characterization of these new enzymes will allow us to uncover more unique biochemical activities.

## References

[ref1] Pedrosa M. C.; Lima L.; Aloso-Esteban J. I.; Roriz C. L.; Barros L.; Ferreira I. C. F. R.; Carocho M. History of Secondary Metabolites: From Ancient Myths to Modern Scientific Validation. Natural Secondary Metabolites: From Nature, Through Science, to Industry 2023, 3–18. 10.1007/978-3-031-18587-8_1.

[ref2] Walsh C. T. Tailoring enzyme strategies and functional groups in biosynthetic pathways. Nat. Prod Rep 2023, 40, 326–386. 10.1039/D2NP00048B. 36268810

[ref3] Tang M. C.; Zou Y.; Watanabe K.; Walsh C. T.; Tang Y. Oxidative Cyclization in Natural Product Biosynthesis. Chem. Rev. 2017, 117, 5226–5333. 10.1021/acs.chemrev.6b00478. 27936626 PMC5406274

[ref4] Walsh C. T.; Tu B. P.; Tang Y. Eight Kinetically Stable but Thermodynamically Activated Molecules that Power Cell Metabolism. Chem. Rev. 2018, 118, 1460–1494. 10.1021/acs.chemrev.7b00510. 29272116 PMC5831524

[ref5] Smanski M. J.; Zhou H.; Claesen J.; Shen B.; Fischbach M. A.; Voigt C. A. Synthetic biology to access and expand nature’s chemical diversity. Nat. Rev. Microbiol 2016, 14, 135–149. 10.1038/nrmicro.2015.24. 26876034 PMC5048682

[ref6] Tibrewal N.; Tang Y. Biocatalysts for natural product biosynthesis. Annu. Rev. Chem. Biomol Eng. 2014, 5, 347–366. 10.1146/annurev-chembioeng-060713-040008. 24910918

[ref7] Fraaije M. W.; Van Berkel W. J. H. Flavin-Containing Oxidative Biocatalysts. In Biocatalysis in the Pharmaceutical and Biotechnology Industries; Patel R. N., Ed.; CRC Press, 2006; pp 181–195

[ref8] Kishimoto S.; Tsunematsu Y.; Sato M.; Watanabe K. Elucidation of Biosynthetic Pathways of Natural Products. Chem. Rec. 2017, 17, 1095–1108. 10.1002/tcr.201700015. 28387469

[ref9] Walsh C. T.; Wencewicz T. A. Flavoenzymes: Versatile catalysts in biosynthetic pathways. Nat. Prod Rep 2013, 30, 175–200. 10.1039/C2NP20069D. 23051833 PMC3518583

[ref10] Fraaije M. W.; Mattevi A. Flavoenzymes: diverse catalysts with recurrent features. Trends Biochem. Sci. 2000, 25, 126–132. 10.1016/S0968-0004(99)01533-9. 10694883

[ref11] Ewing T. A.; Fraaije M. W.; Mattevi A.; Van Berkel W. J. H. The VAO/PCMH flavoprotein family. Arch. Biochem. Biophys. 2017, 632, 104–117. 10.1016/j.abb.2017.06.022. 28669855

[ref12] Fraaije M. W.; Van Berkel W. J. H.; Benen J. A.; Visser J.; Mattevi A. A novel oxidoreductase family sharing a conserved FAD-binding domain. Trends Biochem Sci. 1998, 23, 206–207. 10.1016/S0968-0004(98)01210-9. 9644973

[ref13] Kerschbaumer B.; Bijelic A.; Macheroux P. Flavofun: Exploration of fungal flavoproteomes. Frontiers in Catalysis 2022, 2, 1021691 10.3389/fctls.2022.1021691.

[ref14] Dijkman W. P.; De Gonzalo G.; Mattevi A.; Fraaije M. W. Flavoprotein oxidases: classification and applications. Appl. Microbiol. Biotechnol. 2013, 97, 5177–5188. 10.1007/s00253-013-4925-7. 23640366

[ref15] Mcintire W.; Hopper D. J.; Craig J. C.; Everhart E. T.; Webster R. V.; Causer M. J.; Singer T. P. Stereochemistry of 1-(4’-hydroxyphenyl)ethanol produced by hydroxylation of 4-ethylphenol by p-cresol methylhydroxylase. Biochem. J. 1984, 224, 617 10.1042/bj2240617. 6083780 PMC1144472

[ref16] Fraaije M. W.; Van Den Heuvel R. H. H.; Van Berkel W. J. H.; Mattevi A. Structural analysis of flavinylation in vanillyl-alcohol oxidase. J. Biol. Chem. 2000, 275, 38654–38658. 10.1074/jbc.M004753200. 10984479

[ref17] De Jong E.; Van Berkel W. J. H.; Van der Zwan R. P.; De Bont J. A. M. Purification and characterization of vanillyl-alcohol oxidase from *Penicillium simplicissimum* A novel aromatic alcohol oxidase containing covalently bound FAD. Eur. J. Biochem. 1992, 208, 651–657. 10.1111/j.1432-1033.1992.tb17231.x. 1396672

[ref18] Mattevi A.; Fraaije M. W.; Mozzarelli A.; Olivi L.; Coda A.; van Berkel W. J. H. Crystal structures and inhibitor binding in the octameric flavoenzyme vanillyl-alcohol oxidase: the shape of the active-site cavity controls substrate specificity. Structure 1997, 5, 907–920. 10.1016/S0969-2126(97)00245-1. 9261083

[ref19] Huang C. H.; Lai W. L.; Lee M. H.; Chen C. J.; Vasella A.; Tsai Y. C.; Liaw S. H. Crystal structure of glucooligosaccharide oxidase from *Acremonium strictum*: A novel flavinylation of 6-S-cysteinyl, 8α-N1-histidyl FAD. J. Biol. Chem. 2005, 280, 38831–38838. 10.1074/jbc.M506078200. 16154992

[ref20] Leferink N. G. H.; Heuts D. P. H. M.; Fraaije M. W.; van Berkel W. J. H. The growing VAO flavoprotein family. Arch. Biochem. Biophys. 2008, 474, 292–301. 10.1016/j.abb.2008.01.027. 18280246

[ref21] Heuts D. P. H. M.; Scrutton N. S.; McIntire W. S.; Fraaije M. W. What’s in a covalent bond?: On the role and formation of covalently bound flavin cofactors. FEBS Journal 2009, 276, 3405–3427. 10.1111/j.1742-4658.2009.07053.x. 19438712

[ref22] Ferrari A. R.; Rozeboom H. J.; Dobruchowska J. M.; Van Leeuwen S. S.; Vugts A. S. C.; Koetsier M. J.; Visser J.; Fraaije M. W. Discovery of a xylooligosaccharide oxidase from *Myceliophthora thermophila* C1. J. Biol. Chem. 2016, 291, 23709–23718. 10.1074/jbc.M116.741173. 27629413 PMC5095424

[ref23] Daniel B.; Konrad B.; Toplak M.; Lahham M.; Messenlehner J.; Winkler A.; Macheroux P. The family of berberine bridge enzyme-like enzymes: A treasure-trove of oxidative reactions. Arch. Biochem. Biophys. 2017, 632, 88–103. 10.1016/j.abb.2017.06.023. 28676375

[ref24] Fraaije M. W.; Van Den Heuvel R. H. H.; Van Berkel W. J. H.; Mattevi A. Covalent flavinylation is essential for efficient redox catalysis in vanillyl-alcohol oxidase. J. Biol. Chem. 1999, 274, 35514–35520. 10.1074/jbc.274.50.35514. 10585424

[ref25] Mattevi A. To be or not to be an oxidase: challenging the oxygen reactivity of flavoenzymes. Trends Biochem. Sci. 2006, 31, 276–283. 10.1016/j.tibs.2006.03.003. 16600599

[ref26] Massey V. Activation of molecular oxygen by flavins and flavoproteins. J. Biol. Chem. 1994, 269, 22459–22462. 10.1016/S0021-9258(17)31664-2. 8077188

[ref27] Winkler A.; Łyskowski A.; Riedl S.; Puhl M.; Kutchan T. M.; Macheroux P.; Gruber K. A concerted mechanism for berberine bridge enzyme. Nat. Chem. Biol. 2008, 4, 739–741. 10.1038/nchembio.123. 18953357

[ref28] Zafred D.; Steiner B.; Teufelberger A. R.; Hromic A.; Karplus P. A.; Schofield C. J.; Wallner S.; Macheroux P. Rationally engineered flavin-dependent oxidase reveals steric control of dioxygen reduction. FEBS Journal 2015, 282, 3060–3074. 10.1111/febs.13212. 25619330

[ref29] Gaweska H. M.; Roberts K. M.; Fitzpatrick P. F. Isotope effects suggest a stepwise mechanism for berberine bridge enzyme. Biochemistry 2012, 51, 7342–7347. 10.1021/bi300887m. 22931234 PMC3465707

[ref30] Hawkins K. M.; Smolke C. D. Production of benzylisoquinoline alkaloids in *Saccharomyces cerevisiae*. Nat. Chem. Biol. 2008, 4, 564–573. 10.1038/nchembio.105. 18690217 PMC2830865

[ref31] Daniel B.; Pavkov-Keller T.; Steiner B.; Dordic A.; Gutmann A.; Nidetzky B.; Sensen C. W.; Van Der Graaff E.; Wallner S.; Gruber K.; Macheroux P. Oxidation of monolignols by members of the berberine bridge enzyme family suggests a role in plant cell wall metabolism. J. Biol. Chem. 2015, 290, 18770–18781. 10.1074/jbc.M115.659631. 26037923 PMC4513132

[ref32] Matsumura E.; Nakagawa A.; Tomabechi Y.; Ikushiro S.; Sakaki T.; Katayama T.; Yamamoto K.; Kumagai H.; Sato F.; Minami H. Microbial production of novel sulphated alkaloids for drug discovery. Sci. Rep 2018, 8, 7980 10.1038/s41598-018-26306-7. 29789647 PMC5964154

[ref33] Gao L.; Su C.; Du X.; Wang R.; Chen S.; Zhou Y.; Liu C.; Liu X.; Tian R.; Zhang L.; Xie K.; Chen S.; Guo Q.; Guo L.; Hano Y.; Shimazaki M.; Minami A.; Oikawa H.; Huang N.; Houk K. N.; Huang L.; Dai J.; Lei X. FAD-dependent enzyme-catalysed intermolecular [4 + 2] cycloaddition in natural product biosynthesis. Nat. Chem. 2020, 12, 620–628. 10.1038/s41557-020-0467-7. 32451436

[ref34] Caputi L.; Franke J.; Farrow S. C.; Chung K.; Payne R. M. E.; Nguyen T. D.; Dang T. T. T.; Soares Teto Carqueijeiro I.; Koudounas K.; Dugé De Bernonville T.; Ameyaw B.; Jones D. M.; Curcino Vieira I. J.; Courdavault V.; O’Connor S. E. Missing enzymes in the biosynthesis of the anticancer drug vinblastine in Madagascar periwinkle. Science 2018, 360, 1235–1239. 10.1126/science.aat4100. 29724909

[ref35] Kasahara K.; Miyamoto T.; Fujimoto T.; Oguri H.; Tokiwano T.; Oikawa H.; Ebizuka Y.; Fujii I. Solanapyrone synthase, a possible Diels-Alderase and iterative type I polyketide synthase encoded in a biosynthetic gene cluster from *Alternaria solani*. ChemBioChem. 2010, 11, 1245–1252. 10.1002/cbic.201000173. 20486243

[ref36] Kahlert L.; Cox R. J.; Skellam E. The same but different: multiple functions of the fungal flavin dependent monooxygenase SorD from *Penicillium chrysogenum*. Chem. Commun. 2020, 56, 10934–10937. 10.1039/D0CC03203D. 32789380

[ref37] Liu S. H.; Wei Y. Y.; Xing Y. N.; Chen Y.; Wang W.; Wang K. B.; Liang Y.; Jiao R. H.; Zhang B.; Ge H. M. A BBE-like Oxidase, AsmF, Dictates the Formation of Naphthalenic Hydroxyl Groups in Ansaseomycin Biosynthesis. Org. Lett. 2021, 23, 3724–3728. 10.1021/acs.orglett.1c01101. 33877854

[ref38] Purdy T. N.; Kim M. C.; Cullum R.; Fenical W.; Moore B. S. Discovery and Biosynthesis of Tetrachlorizine Reveals Enzymatic Benzylic Dehydrogenation via an *ortho*-Quinone Methide. J. Am. Chem. Soc. 2021, 143, 3682–3686. 10.1021/jacs.0c12415. 33656337 PMC8318071

[ref39] Yao Y.; An C.; Evans D.; Liu W.; Wang W.; Wei G.; Ding N.; Houk K. N.; Gao S. S. Catalase Involved in Oxidative Cyclization of the Tetracyclic Ergoline of Fungal Ergot Alkaloids. J. Am. Chem. Soc. 2019, 141, 17517–17521. 10.1021/jacs.9b10217. 31621316 PMC7592905

[ref40] Zou Y.; Zhan Z.; Li D.; Tang M.; Cacho R. A.; Watanabe K.; Tang Y. Tandem prenyltransferases catalyze isoprenoid elongation and complexity generation in biosynthesis of quinolone alkaloids. J. Am. Chem. Soc. 2015, 137, 4980–4983. 10.1021/jacs.5b03022. 25859931 PMC4610815

[ref41] Hatch C. E.; Chain W. J. Electrochemically Enabled Total Syntheses of Natural Products. ChemElectroChem. 2023, e202300140 10.1002/celc.202300140. 38106361 PMC10723087

[ref42] Munda M.; Niyogi S.; Shaw K.; Kundu S.; Nandi R.; Bisai A. Electrocatalysis as a key strategy for the total synthesis of natural products. Org. Biomol Chem. 2022, 20, 727–748. 10.1039/D1OB02115J. 34989383

[ref43] Heravi M. M.; Zadsirjan V.; Kouhestanian E.; AlimadadiJani B. Electrochemically Induced Diels-Alder Reaction: An Overview. Chem. Rec. 2020, 20, 273–331. 10.1002/tcr.201900018. 31423739

[ref44] Katayama K.; Kobayashi T.; Oikawa H.; Honma M.; Ichihara A. Enzymatic activity and partial purification of solanapyrone synthase: First enzyme catalyzing Diels-Alder reaction. Biochimica et Biophysica Acta - Protein Structure and Molecular Enzymology 1998, 1384, 387–395. 10.1016/S0167-4838(98)00040-5. 9659400

[ref45] Taura F.; Sirikantaramas S.; Shoyama Y.; Shoyama Y.; Morimoto S. Phytocannabinoids in *Cannabis sativa*: Recent Studies on Biosynthetic Enzymes. Chemistry & Biodiversity 2007, 4, 1649–1663. 10.1002/cbdv.200790145. 17712812

[ref46] Purdy T. N.; Moore B. S.; Lukowski A. L. Harnessing *ortho*-Quinone Methides in Natural Product Biosynthesis and Biocatalysis. J. Nat. Prod 2022, 85, 688 10.1021/acs.jnatprod.1c01026. 35108487 PMC9006567

[ref47] Ames B. D.; Haynes S. W.; Gao X.; Evans B. S.; Kelleher N. L.; Tang Y.; Walsh C. T. Complexity generation in fungal peptidyl alkaloid biosynthesis: Oxidation of fumiquinazoline A to the heptacyclic hemiaminal fumiquinazoline C by the flavoenzyme Af12070 from *Aspergillus fumigatus*. Biochemistry 2011, 50, 8756–8769. 10.1021/bi201302w. 21899262 PMC3194008

[ref48] Chen K. L.; Lai C. Y.; Pham M. T.; Chein R. J.; Tang Y.; Lin H. C. Enzyme-Catalyzed Azepinoindole Formation in Clavine Alkaloid Biosynthesis. Org. Lett. 2020, 22, 3302–3306. 10.1021/acs.orglett.0c01132. 32243182 PMC8092377

[ref49] Nguyen Q. T.; Romero E.; Dijkman W. P.; De Vasconcellos S. P.; Binda C.; Mattevi A.; Fraaije M. W. Structure-Based Engineering of *Phanerochaete chrysosporium* Alcohol Oxidase for Enhanced Oxidative Power toward Glycerol. Biochemistry 2018, 57, 6209–6218. 10.1021/acs.biochem.8b00918. 30272958 PMC6210165

[ref50] Daniel G.; Volc J.; Filonova L.; Plíhal O.; Kubátová E.; Halada P. Characteristics of *Gloeophyllum trabeum* alcohol oxidase, an extracellular source of H_2_O_2_ in brown rot decay of wood. Appl. Environ. Microbiol. 2007, 73, 6241–6253. 10.1128/AEM.00977-07. 17660304 PMC2075019

[ref51] Westrick N. M.; Park S. C.; Keller N. P.; Smith D. L.; Kabbage M. A broadly conserved fungal alcohol oxidase (AOX) facilitates fungal invasion of plants. Mol. Plant Pathol 2023, 24, 28–43. 10.1111/mpp.13274. 36251755 PMC9742500

[ref52] Wong C. M.; Wong K. H.; Chen X. D. Glucose oxidase: Natural occurrence, function, properties and industrial applications. Appl. Microbiol. Biotechnol. 2008, 78, 927 10.1007/s00253-008-1407-4. 18330562

[ref53] Chaerani R.; Voorrips R. E. Tomato early blight (*Alternaria solani*): The pathogen, genetics, and breeding for resistance. Journal of General Plant Pathology 2006, 72, 335–347. 10.1007/s10327-006-0299-3.

[ref54] Auclair K.; Sutherland A.; Kennedy J.; Witter D. J.; Van den Heever J. P.; Hutchinson R. C.; Vederas J. C. Lovastatin Nonaketide Synthase Catalyzes an Intramolecular Diels–Alder Reaction of a Substrate Analogue. J. Am. Chem. Soc. 2000, 122, 11519–11520. 10.1021/ja003216+.

[ref55] Oikawa H.; Katayama K.; Suzuki Y.; Ichihara A. Enzymatic activity catalysing exo-selective Diels-Alder reaction in solanapyrone biosynthesis. J. Chem. Soc. Chem. Commun. 1995, 1321–1322. 10.1039/C39950001321.

[ref56] Wang S.; Wang M.; Duan C.; Yao Y.; Ren J.; Liu L.; Pan Y.; Liu G. A Berberine Bridge Enzyme-like Oxidase Mediates the Cage-like Acresorbicillinol C Biosynthesis. Org. Lett. 2024, 26, 642–646. 10.1021/acs.orglett.3c03966. 38214302

[ref57] Harned A. M.; Volp K. A. The sorbicillinoid family of natural products: Isolation, biosynthesis, and synthetic studies. Nat. Prod Rep 2011, 28, 1790 10.1039/c1np00039j. 21927733

[ref58] Kahlert L.; Bassiony E. F.; Cox R. J.; Skellam E. J. Diels–Alder Reactions During the Biosynthesis of Sorbicillinoids. Angew. Chem. 2020, 132, 5865–5871. 10.1002/ange.201915486. PMC7154774 31943627

[ref59] Guzmán-Chávez F.; Salo O.; Nygård Y.; Lankhorst P. P.; Bovenberg R. A. L.; Driessen A. J. M. Mechanism and regulation of sorbicillin biosynthesis by *Penicillium chrysogenum*. Microb Biotechnol 2017, 10, 958–968. 10.1111/1751-7915.12736. 28618182 PMC5481523

[ref60] Admiraal S. J.; Walsh C. T.; Khosla C. The loading module of rifamycin synthetase is an adenylation - Thiolation didomain with substrate tolerance for substituted benzoates. Biochemistry 2001, 40, 6116–6123. 10.1021/bi010080z. 11352749

[ref61] Xiang P.; Kemmerich B.; Yang L.; Li S. M. Biosynthesis of Annullatin D in *Penicillium roqueforti* Implies Oxidative Lactonization between Two Hydroxyl Groups Catalyzed by a BBE-like Enzyme. Org. Lett. 2022, 24, 6072–6077. 10.1021/acs.orglett.2c02438. 35939524

[ref62] Nies J.; Ran H.; Wohlgemuth V.; Yin W. B.; Li S. M. Biosynthesis of the Prenylated Salicylaldehyde Flavoglaucin Requires Temporary Reduction to Salicyl Alcohol for Decoration before Reoxidation to the Final Product. Org. Lett. 2020, 22, 2256–2260. 10.1021/acs.orglett.0c00440. 32134669

[ref63] Zhao Z.; Ying Y.; Hung Y. S.; Tang Y. Genome Mining Reveals *Neurospora crassa* Can Produce the Salicylaldehyde Sordarial. J. Nat. Prod 2019, 82, 1029–1033. 10.1021/acs.jnatprod.8b00983. 30908040 PMC6933945

[ref64] Liu L.; Tang M. C.; Tang Y. Fungal highly reducing polyketide synthases biosynthesize salicylaldehydes that are precursors to epoxycyclohexenol natural products. J. Am. Chem. Soc. 2019, 141, 19538–19541. 10.1021/jacs.9b09669. 31790246 PMC6924165

[ref65] Cowled M. S.; Li H.; Gilchrist C. L. M.; Lacey E.; Chooi Y. H.; Piggott A. M. Stereodivergent Hydroxylation of Berkeleylactones by *Penicillium turbatum*. J. Nat. Prod 2023, 86, 541–549. 10.1021/acs.jnatprod.2c00946. 36524608

[ref66] Zhang Y.; Bai J.; Zhang L.; Zhang C.; Liu B.; Hu Y. Self-Resistance in the Biosynthesis of Fungal Macrolides Involving Cycles of Extracellular Oxidative Activation and Intracellular Reductive Inactivation. Angew. Chem. 2021, 60, 6639–6645. 10.1002/anie.202015442. 33314510

[ref67] Stierle A. A.; Stierle D. B.; Decato D.; Priestley N. D.; Alverson J. B.; Hoody J.; McGrath K.; Klepacki D. The Berkeleylactones, Antibiotic Macrolides from Fungal Coculture. J. Nat. Prod. 2017, 80, 1150–1160. 10.1021/acs.jnatprod.7b00133. 28326781 PMC5467647

[ref68] Kim J.-E.; Son H.; Lee Y.-W. Biosynthetic mechanism and regulation of zearalenone in *Fusarium graminearum*. JSM Mycotoxins 2018, 68, 1–6. 10.2520/myco.68-1-2.

[ref69] Fu P.; Wang S.; Hong K.; Li X.; Liu P.; Wang Y.; Zhu W. Cytotoxic bipyridines from the marine-derived actinomycete *Actinoalloteichus cyanogriseus* WH1–2216–6. J. Nat. Prod 2011, 74, 1751–1756. 10.1021/np200258h. 21770434

[ref70] Zhu Y.; Picard M. È.; Zhang Q.; Barma J.; Després X. M.; Mei X.; Zhang L.; Duvignaud J. B.; Couture M.; Zhu W.; Shi R.; Zhang C. Flavoenzyme CrmK-mediated substrate recycling in caerulomycin biosynthesis. Chem. Sci. 2016, 7, 4867–4874. 10.1039/C6SC00771F. 30155134 PMC6016722

[ref71] Luzhetskyy A.; Fedoryshyn M.; Dürr C.; Taguchi T.; Novikov V.; Bechthold A. Iteratively acting glycosyltransferases involved in the hexasaccharide biosynthesis of landomycin A. Chem. Biol. 2005, 12, 725–729. 10.1016/j.chembiol.2005.05.008. 16039521

[ref72] Goda S. K.; Akhtar M. Neomycin biosynthesis: The incorporation of d-6-deoxy-glucose derivatives and variously labelled glucose into the 2-deoxystreptamine ring: Postulated involvement of 2-deoxyinosose synthase in the biosynthesis. J. Antibiot (Tokyo) 1992, 45, 984–994. 10.7164/antibiotics.45.984. 1500367

[ref73] Rohr J.; Wohlert S.-E.; Oelkers C.; Kirschning A.; Ries M. Biosynthetic short activation of the 2,3,6-trideoxysugar l-rhodinose. Chem. Commun. 1997, 2, 973–974. 10.1039/a702125i.

[ref74] Chen L.; Wang X.; Zou Y.; Tang M. C. Genome Mining of a Fungal Polyketide Synthase-Nonribosomal Peptide Synthetase Hybrid Megasynthetase Pathway to Synthesize a Phytotoxic N-Acyl Amino Acid. Org. Lett. 2024, 26, 3597–3601. 10.1021/acs.orglett.4c01039. 38661293

[ref75] Boettger D.; Hertweck C. Molecular Diversity Sculpted by Fungal PKS-NRPS Hybrids. ChemBioChem. 2013, 14, 28–42. 10.1002/cbic.201200624. 23225733

[ref76] Li Y. S.; Ho J. Y.; Huang C. C.; Lyu S. Y.; Lee C. Y.; Huang Y. T.; Wu C. J.; Chan H. C.; Huang C. J.; Hsu N. S.; Tsai M. D.; Li T. L. A unique flavin mononucleotide-linked primary alcohol oxidase for glycopeptide A40926 maturation. J. Am. Chem. Soc. 2007, 129, 13384–13385. 10.1021/ja075748x. 17935335

[ref77] Liu Y. C.; Li Y. S.; Lyu S. Y.; Hsu L. J.; Chen Y. H.; Huang Y. T.; Chan H. C.; Huang C. J.; Chen G. H.; Chou C. C.; Tsai M. D.; Li T. L. Interception of teicoplanin oxidation intermediates yields new antimicrobial scaffolds. Nat. Chem. Biol. 2011, 7, 304–309. 10.1038/nchembio.556. 21478878

[ref78] Borch R. F.; Bernstein M. D.; Durst H. D. Cyanohydridoborate anion as a selective reducing agent. J. Am. Chem. Soc. 1971, 93, 2897–2904. 10.1021/ja00741a013.

[ref79] Alexeev I.; Sultana A.; Mäntsälä P.; Niemi J.; Schneider G. Aclacinomycin oxidoreductase (AknOx) from the biosynthetic pathway of the antibiotic aclacinomycin is an unusual flavoenzyme with a dual active site. Proc. Natl. Acad. Sci. U. S. A. 2007, 104, 6170–6175. 10.1073/pnas.0700579104. 17395717 PMC1851095

[ref80] Eckardt K.; Wagner C. Biosynthesis of anthracyclinones. J. Basic Microbiol 1988, 28, 137–144. 10.1002/jobm.3620280117. 3171921

[ref81] Zhang Y.; Huang H.; Chen Q.; Luo M.; Sun A.; Song Y.; Ma J.; Ju J. Identification of the grincamycin gene cluster unveils divergent roles for GcnQ in different hosts, tailoring the L-rhodinose moiety. Org. Lett. 2013, 15, 3254–3257. 10.1021/ol401253p. 23782455

[ref82] Carlson J. C.; Fortman J. L.; Anzai Y.; Li S.; Burr D. A.; Sherman D. H. Identification of the tirandamycin biosynthetic gene cluster from *Streptomyces* sp. 307–9. ChemBioChem. 2010, 11, 564–572. 10.1002/cbic.200900658. 20127927 PMC3019614

[ref83] Carlson J. C.; Li S.; Gunatilleke S. S.; Anzai Y.; Burr D. A.; Podust L. M.; Sherman D. H. Tirandamycin biosynthesis is mediated by co-dependent oxidative enzymes. Nat. Chem. 2011, 3, 628–633. 10.1038/nchem.1087. 21778983 PMC3154026

[ref84] Reusser F. Tirandamycin, an Inhibitor of Bacterial Ribonucleic Acid Polymerase. Antimicrob. Agents Chemother. 1976, 10, 618–622. 10.1128/AAC.10.4.618. 791108 PMC429803

[ref85] Carlson J. C.; Li S.; Burr D. A.; Sherman D. H. Isolation and characterization of tirandamycins from a marine-derived Streptomyces sp. J. Nat. Prod 2009, 72, 2076 10.1021/np9005597. 19883065 PMC2873692

[ref86] Kharel M. K.; Pahari P.; Lian H.; Rohr J. GilR, an unusual lactone-forming enzyme involved in gilvocarcin biosynthesis. ChemBioChem. 2009, 10, 1305–1308. 10.1002/cbic.200900130. 19388008 PMC2879341

[ref87] Knobler R. M.; Radlwimmer F. B.; Lane M. J. Gilvocarcin V exhibits both equilibrium DNA binding and UV light induced DNA adduct formation which is sequence context dependent. Nucleic Acids Res. 1992, 20, 4553–4557. 10.1093/nar/20.17.4553. 1408756 PMC334184

[ref88] Cai X.; Ng K.; Panesar H.; Moon S. J.; Paredes M.; Ishida K.; Hertweck C.; Minehan T. G. Total synthesis of the antitumor natural product polycarcin v and evaluation of its DNA binding profile. Org. Lett. 2014, 16, 2962–2965. 10.1021/ol501095w. 24824354 PMC4059221

[ref89] Noinaj N.; Bosserman M. A.; Schickli M. A.; Piszczek G.; Kharel M. K.; Pahari P.; Buchanan S. K.; Rohr J. The crystal structure and mechanism of an unusual oxidoreductase, GilR, involved in gilvocarcin V biosynthesis. J. Biol. Chem. 2011, 286, 23533–23543. 10.1074/jbc.M111.247833. 21561854 PMC3123116

[ref90] Shoyama Y.; Tamada T.; Kurihara K.; Takeuchi A.; Taura F.; Arai S.; Blaber M.; Shoyama Y.; Morimoto S.; Kuroki R. Structure and function of Δ1-tetrahydrocannabinolic acid (THCA) synthase, the enzyme controlling the psychoactivity of *Cannabis sativa*. J. Mol. Biol. 2012, 423, 96–105. 10.1016/j.jmb.2012.06.030. 22766313

[ref91] Gonçalves J.; Rosado T.; Soares S.; Simão A.; Caramelo D.; Luís Â.; Fernández N.; Barroso M.; Gallardo E.; Duarte A. Cannabis and Its Secondary Metabolites: Their Use as Therapeutic Drugs, Toxicological Aspects, and Analytical Determination. Medicines 2019, 6, 31–78. 10.3390/medicines6010031. 30813390 PMC6473697

[ref92] Liu Y.; Jing S. X.; Luo S. H.; Li S. H. Non-volatile natural products in plant glandular trichomes: Chemistry, biological activities and biosynthesis. Nat. Prod Rep 2019, 36, 626 10.1039/C8NP00077H. 30468448

[ref93] Pollastro F.; Caprioglio D.; Del Prete D.; Rogati F.; Minassi A.; Taglialatela-Scafati O.; Munoz E.; Appendino G. Cannabichromene. Nat. Prod Commun. 2018, 13, 1189–1194. 10.1177/1934578X1801300922.

[ref94] Mantovani S. M.; Moore B. S. Flavin-linked oxidase catalyzes pyrrolizine formation of dichloropyrrole-containing polyketide extender unit in chlorizidine a. J. Am. Chem. Soc. 2013, 135, 18032–18035. 10.1021/ja409520v. 24246014 PMC3887146

[ref95] Alvarez-Mico X.; Jensen P. R.; Fenical W.; Hughes C. C. Chlorizidine, a cytotoxic 5H-pyrrolo[2,1-a]isoindol-5-one-containing alkaloid from a marine *Streptomyces* sp. Org. Lett. 2013, 15, 988–991. 10.1021/ol303374e. 23405849 PMC3702164

[ref96] Nomura T.; Hano Y.; Fukai T. Chemistry and biosynthesis of isoprenylated flavonoids from Japanese mulberry tree. Proc. Jpn. Acad. Ser. B Phys. Biol. Sci. 2009, 85, 391 10.2183/pjab.85.391. PMC3621561 19907125

[ref97] Zou Y.; Garcia-Borràs M.; Tang M. C.; Hirayama Y.; Li D. H.; Li L.; Watanabe K.; Houk K. N.; Tang Y. Enzyme-catalyzed cationic epoxide rearrangements in quinolone alkaloid biosynthesis. Nat. Chem. Biol. 2017, 13, 325–332. 10.1038/nchembio.2283. 28114276 PMC5310975

[ref98] Ishikawa N.; Tanaka H.; Koyama F.; Noguchi H.; Wang C. C. C.; Hotta K.; Watanabe K. Non-Heme Dioxygenase Catalyzes Atypical Oxidations of 6,7-Bicyclic Systems to Form the 6,6-Quinolone Core of Viridicatin-Type Fungal Alkaloids. Angewandte Chemie - International Edition 2014, 53, 12880–12884. 10.1002/anie.201407920. 25251934

[ref99] Kishimoto S.; Hara K.; Hashimoto H.; Hirayama Y.; Champagne P. A.; Houk K. N.; Tang Y.; Watanabe K. Enzymatic one-step ring contraction for quinolone biosynthesis. Nat. Commun. 2018, 9, 2826 10.1038/s41467-018-05221-5. 30026518 PMC6053404

[ref100] Klas K. R.; Kato H.; Frisvad J. C.; Yu F.; Newmister S. A.; Fraley A. E.; Sherman D. H.; Tsukamoto S.; Williams R. M. Structural and stereochemical diversity in prenylated indole alkaloids containing the bicyclo[2.2.2]diazaoctane ring system from marine and terrestrial fungi. Nat. Prod Rep 2018, 35, 532–558. 10.1039/C7NP00042A. 29632911 PMC6102066

[ref101] Li S.; Srinivasan K.; Tran H.; Yu F.; Finefield J. M.; Sunderhaus J. D.; McAfoos T. J.; Tsukamoto S.; Williams R. M.; Sherman D. H. Comparative analysis of the biosynthetic systems for fungal bicyclo[2.2.2]diazaoctane indole alkaloids: The (+)/(−)-notoamide, paraherquamide and malbrancheamide pathways. Medchemcomm 2012, 3, 987–996. 10.1039/c2md20029e. 23213353 PMC3511817

[ref102] Sunderhaus J. D.; McAfoos T. J.; Finefield J. M.; Kato H.; Li S.; Tsukamoto S.; Sherman D. H.; Williams R. M. Synthesis and bioconversions of notoamide T: A biosynthetic precursor to stephacidin A and notoamide B. Org. Lett. 2013, 15, 22–25. 10.1021/ol302901p. 23249380 PMC3549551

[ref103] Hu J.; Sarrami F.; Li H.; Zhang G.; Stubbs K. A.; Lacey E.; Stewart S. G.; Karton A.; Piggott A. M.; Chooi Y. H. Heterologous biosynthesis of elsinochrome A sheds light on the formation of the photosensitive perylenequinone system. Chem. Sci. 2019, 10, 1457–1465. 10.1039/C8SC02870B. 30809363 PMC6354827

[ref104] De Jonge R.; Ebert M. K.; Huitt-Roehl C. R.; Pal P.; Suttle J. C.; Spanner R. E.; Neubauer J. D.; Jurick W. M.; Stott K. A.; Secor G. A.; Thomma B. P. H. J.; Van De Peer Y.; Townsend C. A.; Bolton M. D. Gene cluster conservation provides insight into cercosporin biosynthesis and extends production to the genus Colletotrichum. Proc. Natl. Acad. Sci. U. S. A. 2018, 115, E5459–E5466. 10.1073/pnas.1712798115. 29844193 PMC6004482

[ref105] Kutchan T. M.; Dittrich H. Characterization and Mechanism of the Berberine Bridge Enzyme, a Covalently Flavinylated Oxidase of Benzophenanthridine Alkaloid Biosynthesis in Plants. J. Biol. Chem. 1995, 270, 24475–24481. 10.1074/jbc.270.41.24475. 7592663

[ref106] Amann M.; Nagakura N.; Zenk M. H. Purification and properties of (S)-tetrahydroprotoberberine oxidase from suspension-cultured cells of *Berberis wilsoniae*. Eur. J. Biochem. 1988, 175, 17–25. 10.1111/j.1432-1033.1988.tb14160.x. 3402447

[ref107] Chou W. M.; Kutchan T. M. Enzymatic oxidations in the biosynthesis of complex alkaloids. Plant Journal 1998, 15, 289–300. 10.1046/j.1365-313X.1998.00220.x. 9750342

[ref108] Gesell A.; Diaz Chávez M. L.; Kramell R.; Piotrowski M.; Macheroux P.; Kutchan T. M. Heterologous expression of two FAD-dependent oxidases with (S)-tetrahydroprotoberberine oxidase activity from *Argemone mexicana* and *Berberis wilsoniae* in insect cells. Planta 2011, 233, 1185–1197. 10.1007/s00425-011-1357-4. 21327819

[ref109] Winkler A.; Puhl M.; Weber H.; Kutchan T. M.; Gruber K.; Macheroux P. Berberine bridge enzyme catalyzes the six-electron oxidation of (S)-reticuline to dehydroscoulerine. Phytochemistry 2009, 70, 1092–1097. 10.1016/j.phytochem.2009.06.005. 19570558

[ref110] Qu Y.; Safonova O.; De Luca V. Completion of the canonical pathway for assembly of anticancer drugs vincristine/vinblastine in *Catharanthus roseus*. Plant Journal 2019, 97, 257–266. 10.1111/tpj.14111. 30256480

[ref111] Fan Z.; Jaisi A.; Chen Y.; Shen L.; Liu Z.; Wu S.; Liu Y.; Zhang W.; Xiao Y. Discovery and Biosynthesis of Ascorbylated Securinega Alkaloids. ACS Catal. 2021, 11, 8818–8828. 10.1021/acscatal.1c01514.

[ref112] Kang G.; Park S.; Han S. The Chemistry of High-Oxidation State Securinega Alkaloids. Eur. J. Org. Chem. 2021, 2021, 1508–1520. 10.1002/ejoc.202001610.

[ref113] Wehlauch R.; Gademann K. Securinega Alkaloids: Complex Structures, Potent Bioactivities, and Efficient Total Syntheses. Asian J. Org. Chem. 2017, 6, 1146–1159. 10.1002/ajoc.201700142.

[ref114] Song L. Q.; Zhang Y. Y.; Pu J. Y.; Tang M. C.; Peng C.; Tang G. L. Catalysis of Extracellular Deamination by a FAD-Linked Oxidoreductase after Prodrug Maturation in the Biosynthesis of Saframycin A. Angewandte Chemie - International Edition 2017, 56, 9116–9120. 10.1002/anie.201704726. 28561936

[ref115] Zhang Y.; Wen W. H.; Pu J. Y.; Tang M. C.; Zhang L.; Peng C.; Xu Y.; Tang G. L. Extracellularly oxidative activation and inactivation of matured prodrug for cryptic self-resistance in naphthyridinomycin biosynthesis. Proc. Natl. Acad. Sci. U. S. A. 2018, 115, 11232–11237. 10.1073/pnas.1800502115. 30327344 PMC6217432

[ref116] Fitzpatrick P. F. Oxidation of amines by flavoproteins. Arch. Biochem. Biophys. 2010, 493, 13–25. 10.1016/j.abb.2009.07.019. 19651103 PMC2812625

[ref117] Tang S.; Zhang W.; Li Z.; Li H.; Geng C.; Huang X.; Lu X. Discovery and Characterization of a PKS-NRPS Hybrid in *Aspergillus terreus* by Genome Mining. J. Nat. Prod 2020, 83, 473–480. 10.1021/acs.jnatprod.9b01140. 32077283

[ref118] Yamamoto T.; Tsunematsu Y.; Noguchi H.; Hotta K.; Watanabe K. Elucidation of Pyranonigrin Biosynthetic Pathway Reveals a Mode of Tetramic Acid, Fused γ-Pyrone, and exo-Methylene Formation. Org. Lett. 2015, 17, 4992–4995. 10.1021/acs.orglett.5b02435. 26414728

[ref119] Rocha M. C.; Fabri J. H. T. M.; Silva L. P.; Angolini C. F. F.; Bertolini M. C.; da Cunha A. F.; Valiante V.; Goldman G. H.; Fill T. P.; Malavazi I. Transcriptional Control of the Production of *Aspergillus fumigatus* Conidia-Borne Secondary Metabolite Fumiquinazoline C Important for Phagocytosis Protection. Genetics 2021, 218, iyab036 10.1093/genetics/iyab036. 33705521 PMC9335938

[ref120] Lim F. Y.; Ames B.; Walsh C. T.; Keller N. P. Co-ordination between BrlA regulation and secretion of the oxidoreductase FmqD directs selective accumulation of fumiquinazoline C to conidial tissues in *Aspergillus fumigatus*. Cell Microbiol 2014, 16, 1267–1283. 10.1111/cmi.12284. 24612080 PMC4114987

[ref121] Mantegani S.; Brambilla E.; Varasi M. Ergoline derivatives: Receptor affinity and selectivity. Farmaco 1999, 54, 288–296. 10.1016/S0014-827X(99)00028-2. 10418123

[ref122] Sharma N.; Sharma V.; Manikyam H.; Krishna A. Ergot Alkaloids: A Review on Therapeutic Applications. European J. Med. Plants 2016, 14, 1–17. 10.9734/EJMP/2016/25975.

[ref123] Pertz H.; Eich E. Ergot Alkaloids and their Derivatives as Ligands for Serotoninergic, Dopaminergic, and Adrenergic Receptors. Ergot: The Genus Claviceps; Křen V., Cvak L., Eds.; Harwood Academic Publishers: Amsterdam, 1999; pp 411–440.

[ref124] Wong G.; Lim L. R.; Tan Y. Q.; Go M. K.; Bell D. J.; Freemont P. S.; Yew W. S. Reconstituting the complete biosynthesis of D-lysergic acid in yeast. Nat. Commun. 2022, 13, 712 10.1038/s41467-022-28386-6. 35132076 PMC8821704

[ref125] Lin H. C.; Chiou G.; Chooi Y. H.; McMahon T. C.; Xu W.; Garg N. K.; Tang Y. Elucidation of the concise biosynthetic pathway of the communesin indole alkaloids. Angewandte Chemie - International Edition 2015, 54, 3004–3007. 10.1002/anie.201411297. 25571861 PMC4409825

[ref126] Lorenz N.; Olsovska J.; Šulc S.; Tudzynski P. Alkaloid Cluster Gene ccsA of the Ergot Fungus *Claviceps purpurea* Encodes Chanoclavine I Synthase, a Flavin Adenine Dinucleotide-Containing Oxidoreductase Mediating the Transformation of N-Methyl-Dimethylallyltryptophan. Appl. Environ. Microbiol. 2010, 76, 1822–1830. 10.1128/AEM.00737-09. 20118373 PMC2838005

[ref127] Reddy P.; Guthridge K.; Vassiliadis S.; Hemsworth J.; Hettiarachchige I.; Spangenberg G.; Rochfort S. Tremorgenic mycotoxins: Structure diversity and biological activity. Toxins (Basel) 2019, 11, 302 10.3390/toxins11050302. 31137882 PMC6563255

[ref128] Hou Y.; Chen M.; Sun Z.; Ma G.; Chen D.; Wu H.; Yang J.; Li Y.; Xu X. The Biosynthesis Related Enzyme, Structure Diversity and Bioactivity Abundance of Indole-Diterpenes: A Review. Molecules 2022, 27, 6870 10.3390/molecules27206870. 36296463 PMC9611320

[ref129] Wei X.; Wang W. G.; Matsuda Y. Branching and converging pathways in fungal natural product biosynthesis. Fungal Biol. Biotechnol 2022, 9, 6 10.1186/s40694-022-00135-w. 35255990 PMC8902786

[ref130] Ozaki T.; Minami A.; Oikawa H. Biosynthesis of indole diterpenes: a reconstitution approach in a heterologous host. Nat. Prod Rep 2023, 40, 202–213. 10.1039/D2NP00031H. 36321441

[ref131] Liu Y.; Ozaki T.; Minami A.; Oikawa H. Oxidative bicyclic ring system formation involving indole diterpene biosynthesis: Remarkable substrate tolerance of a prenyltransferase and flavoprotein oxidase. Tetrahedron Lett. 2023, 117, 154374 10.1016/j.tetlet.2023.154374.

[ref132] Liu C.; Minami A.; Dairi T.; Gomi K.; Scott B.; Oikawa H. Biosynthesis of Shearinine: Diversification of a Tandem Prenyl Moiety of Fungal Indole Diterpenes. Org. Lett. 2016, 18, 5026–5029. 10.1021/acs.orglett.6b02482. 27632559

[ref133] Nicholson M. J.; Eaton C. J.; Stärkel C.; Tapper B. A.; Cox M. P.; Scott B. Molecular cloning and functional analysis of gene clusters for the biosynthesis of indole-diterpenes in *Penicillium crustosum* and *P. Janthinellum*. Toxins (Basel) 2015, 7, 2701–2722. 10.3390/toxins7082701. 26213965 PMC4549719

[ref134] Van De Bittner K. C.; Nicholson M. J.; Bustamante L. Y.; Kessans S. A.; Ram A.; Van Dolleweerd C. J.; Scott B.; Parker E. J. Heterologous Biosynthesis of Nodulisporic Acid F. J. Am. Chem. Soc. 2018, 140, 582–585. 10.1021/jacs.7b10909. 29283570

[ref135] Richardson A. T.; Cameron R. C.; Stevenson L. J.; Singh A. J.; Lukito Y.; Berry D.; Nicholson M. J.; Parker E. J. Biosynthesis of Nodulisporic Acids: A Multifunctional Monooxygenase Delivers a Complex and Highly Branched Array. Angewandte Chemie - International Edition 2022, 61, e202213364 10.1002/anie.202213364. 36199176 PMC10098816

[ref136] Liu C.; Tagami K.; Minami A.; Matsumoto T.; Frisvad J. C.; Suzuki H.; Ishikawa J.; Gomi K.; Oikawa H. Reconstitution of biosynthetic machinery for the synthesis of the highly elaborated indole diterpene penitrem. Angewandte Chemie - International Edition 2015, 54, 5748–5752. 10.1002/anie.201501072. 25831977

[ref137] Gao L.; Yang J.; Lei X. Enzymatic intermolecular Diels-Alder reactions in synthesis: From nature to design. Tetrahedron Chem. 2022, 2, 100013 10.1016/j.tchem.2022.100013.

[ref138] Gao L.; Zou Y.; Liu X.; Yang J.; Du X.; Wang J.; Yu X.; Fan J.; Jiang M.; Li Y.; Houk K. N.; Lei X. Enzymatic control of endo- and exo-stereoselective Diels–Alder reactions with broad substrate scope. Nat. Catal 2021, 4, 1059–1069. 10.1038/s41929-021-00717-8.

[ref139] Ding Q.; Guo N.; Gao L.; McKee M.; Wu D.; Yang J.; Fan J.; Weng J. K.; Lei X. The evolutionary origin of naturally occurring intermolecular Diels-Alderases from *Morus alba*. Nat. Commun. 2024, 15, 2492 10.1038/s41467-024-46845-0. 38509059 PMC10954736

[ref140] Li H.; Hu J.; Wei H.; Solomon P. S.; Stubbs K. A.; Chooi Y. H. Biosynthesis of a Tricyclo[6.2.2.02,7]dodecane System by a Berberine Bridge Enzyme-Like Aldolase. Chem. Eur. J. 2019, 25, 15062–15066. 10.1002/chem.201904360. 31553484

[ref141] Ugai T.; Minami A.; Fujii R.; Tanaka M.; Oguri H.; Gomi K.; Oikawa H. Heterologous expression of highly reducing polyketide synthase involved in betaenone biosynthesis. Chem. Commun. 2015, 51, 1878–1881. 10.1039/C4CC09512J. 25530455

[ref142] Chiang Y. M.; Lin T. S.; Wang C. C. C. Total Heterologous Biosynthesis of Fungal Natural Products in *Aspergillus nidulans*. J. Nat. Prod 2022, 85, 2484–2518. 10.1021/acs.jnatprod.2c00487. 36173392 PMC9621686

[ref143] Zhang J. M.; Liu X.; Wei Q.; Ma C.; Li D.; Zou Y. Berberine bridge enzyme-like oxidase-catalysed double bond isomerization acts as the pathway switch in cytochalasin synthesis. Nat. Commun. 2022, 13, 225 10.1038/s41467-021-27931-z. 35017571 PMC8752850

[ref144] Huang X.; Zhang W.; Tang S.; Wei S.; Lu X. Collaborative Biosynthesis of a Class of Bioactive Azaphilones by Two Separate Gene Clusters Containing Four PKS/NRPSs with Transcriptional Crosstalk in Fungi. Angewandte Chemie - International Edition 2020, 59, 4349–4353. 10.1002/anie.201915514. 31908094

[ref145] Heard S. C.; Wu G.; Winter J. M. Discovery and characterization of a cytochalasan biosynthetic cluster from the marine-derived fungus *Aspergillus flavipes* CNL-338. J. Antibiot. 2020, 73, 803–807. 10.1038/s41429-020-00368-0. 32913332

[ref146] Li H.; Wei H.; Hu J.; Lacey E.; Sobolev A. N.; Stubbs K. A.; Solomon P. S.; Chooi Y. H. Genomics-Driven Discovery of Phytotoxic Cytochalasans Involved in the Virulence of the Wheat Pathogen *Parastagonospora nodorum*. ACS Chem. Biol. 2020, 15, 226–233. 10.1021/acschembio.9b00791. 31815421

[ref147] Zhang X.; Pang X.; Zhang L.; Li Y.; Song Y.; Xiao H.; Liu Y.; Wang J.; Yan Y. Genome Mining Uncovers a Flavoenzyme-Catalyzed Isomerization Process during the Maturation of Pyrenophorol Dilactones. Org. Lett. 2024, 26, 1612–1617. 10.1021/acs.orglett.4c00008. 38377309

[ref148] Teufel R.; Miyanaga A.; Michaudel Q.; Stull F.; Louie G.; Noel J. P.; Baran P. S.; Palfey B.; Moore B. S. Flavin-mediated dual oxidation controls an enzymatic Favorskii-type rearrangement. Nature 2013, 503, 552–556. 10.1038/nature12643. 24162851 PMC3844076

[ref149] Teufel R.; Stull F.; Meehan M. J.; Michaudel Q.; Dorrestein P. C.; Palfey B.; Moore B. S. Biochemical Establishment and Characterization of EncM’s Flavin-N5-oxide Cofactor. J. Am. Chem. Soc. 2015, 137, 8078–8085. 10.1021/jacs.5b03983. 26067765 PMC4720136

[ref150] Beaupre B. A.; Moran G. R. N5 Is the New C4a: Biochemical Functionalization of Reduced Flavins at the N5 Position. Front Mol. Biosci 2020, 7, 598912 10.3389/fmolb.2020.598912. 33195440 PMC7662398

